# Role of Nitrogenous Functional Group Identity in Accelerating 1,2,3-Trichloropropane Degradation by Pyrogenic Carbonaceous Matter (PCM) and Sulfide Using PCM-like Polymers

**DOI:** 10.1021/acs.est.3c11010

**Published:** 2024-06-07

**Authors:** Han Cao, Jingdong Mao, Paul G. Tratnyek, Wenqing Xu

**Affiliations:** † Department of Civil and Environmental Engineering, Villanova University, Villanova, Pennsylvania 19085, United States; ‡ Department of Chemistry and Biochemistry, Old Dominion University, Norfolk, Virginia 23529, United States; § OHSU/PSU School of Public Health, Oregon Health & Science University, 3181 SW Sam Jackson Park Road, Portland, Oregon 97239, United States

**Keywords:** volatile organic contaminant (VOC), chlorinated solvents, nitrogenous functional groups, quaternary ammonium, pyridinium cation, sulfide, nucleophilic substitution, ß-elimination, biochar

## Abstract

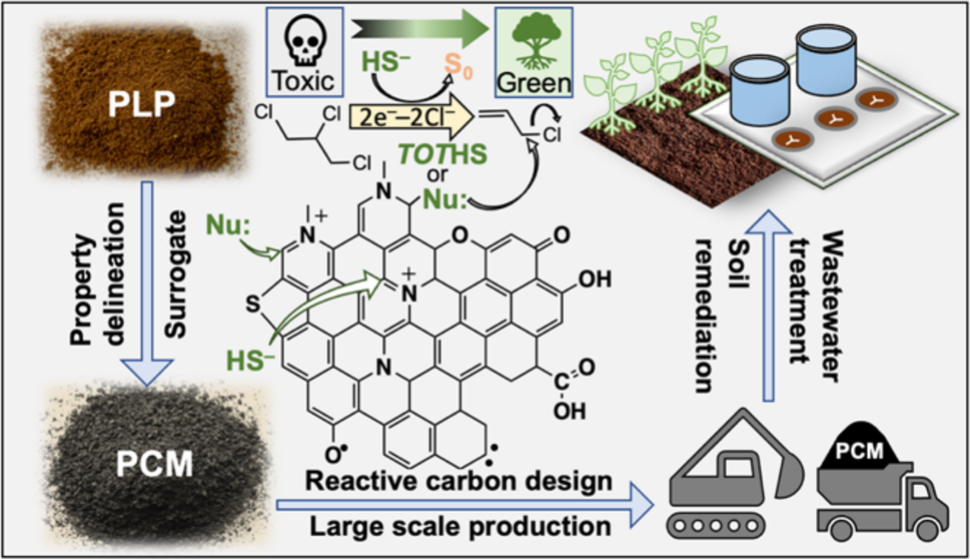

Groundwater contamination by 1,2,3-trichloropropane (TCP) poses a unique challenge due to its human toxicity and recalcitrance to degradation. Previous work suggests that nitrogenous functional groups of pyrogenic carbonaceous matter (PCM), such as biochar, are important in accelerating contaminant dechlorination by sulfide. However, the reaction mechanism is unclear due, in part, to PCM’s structural complexity. Herein, PCM-like polymers (PLPs) with controlled placement of nitrogenous functional groups [i.e., quaternary ammonium (QA), pyridine, and pyridinium cations (py^+^)] were employed as model systems to investigate PCM-enhanced TCP degradation by sulfide. Our results suggest that both PLP-QA and PLP-py^+^ were highly effective in facilitating TCP dechlorination by sulfide with half-lives of 16.91 ± 1.17 and 0.98 ± 0.15 days, respectively, and the reactivity increased with surface nitrogenous group density. A two-step process was proposed for TCP dechlorination, which is initiated by reductive ß-elimination, followed by nucleophilic substitution by surface-bound sulfur nucleophiles. The TCP degradation kinetics were not significantly affected by cocontaminants (i.e., 1,1,1-trichloroethane or trichloroethylene), but were slowed by natural organic matter. Our results show that PLPs containing certain nitrogen functional groups can facilitate the rapid and complete degradation of TCP by sulfide, suggesting that similarly functionalized PCM might form the basis for a novel process for the remediation of TCP-contaminated groundwater.

## Abstract 2

Pyrogenic carbonaceous matter decorated with quaternary ammonium and pyridinium cation groups significantly enhanced the degradation to complete dechlorination of 1,2,3-trichloropropane by sulfide.

## Introduction

1,2,3-Trichloropropane (TCP) emerges as an environmental contaminant through various sources, including cleaning or degreasing solvents, chemical manufacturing (e.g., of epichlorohydrin), and agricultural use of certain fumigants.^1−3^ As a result, TCP contamination of groundwater can occur by point and nonpoint source scenarios, with the latter resulting in groundwater TCP concentrations up to 100 μg·L^–1^.^4−6^ TCP has been classified as “Group 2A-probably carcinogenic to humans” by the International Agency for Research on Cancer (IARC). In addition, it exhibits higher toxicity (e.g., lower oral reference dose) compared with other chlorinated solvents (e.g., 1,1,1-trichloroethane or 1,1-dichloroethylene). Consequently, significant health effects can be expected even from lower exposures.^2,7^ TCP is currently on the EPA’s Contaminant Candidate List 5 (CCL-5) and is regulated by the states of California, New Jersey, and Hawaii, where the current maximum contaminant levels (MCL) are 5, 30, and 600 ng·L^–1^, respectively.^8^ Due to its moderate mobility (log *K*_oc_ = 1.70–1.99) and resistance to environmental degradation, TCP can migrate into groundwater, where it is highly persistent with an estimated half-life of up to 77 years.^7,8^

Recently, various technologies have been developed for TCP remediation.^1,2,9−16^ The most common treatment is (*ex situ*) adsorption to granular activated carbon (GAC), which can achieve up to 99% removal of TCP to a level below 5 ng·L^–1^.^16^ However, TCP adsorption by GAC is less favorable than that of many other organics and requires frequent and costly replacement of the adsorbent.^7,8,17^ For instance, the estimated annual cost of GAC for TCP removal was over $33 million for a large water system [>1 million gallons per day (MGD)].^18,19^ Biotic degradation of TCP can occur; for example, bacteria containing haloalkane dehalogenase have been shown to mineralize TCP.^13,14^ However, studies suggest that bioremediation can sometimes generate byproducts like allyl chloride and dichloropropanol, which are more toxic than the parent compound TCP.^12−15,20^ Abiotic reductive dechlorination of TCP with zerovalent metals such as zerovalent zinc (ZVZ) can achieve complete dechlorination to products such as propane or propene with a half-life of several hours.^1,2,11^ However, the reactivity of ZVZ is strongly influenced by the (de)passivation processes, which is not well understood and therefore difficult to navigate in the field applications.^2^ Advanced oxidation processes (AOPs), such as the Fenton reaction, tend to generate toxic byproducts such as 1,3-dichloro-2-propanone and 2,3-dichloro-1-propene, which are more problematic.^9,10^ Overall, there is a need for more efficient, cost-effective, and sustainable technologies for the remediation of TCP-contaminated groundwater.

Biochar has recently been shown to accelerate the dechlorination of chlorinated solvents by sulfide, including trichloroethylene, tetrachloroethylene, hexachloroethane, and carbon tetrachloride.^21−24^ Based on the positive correlation between observed rate constants (*k*_obs_) and the pyridinic nitrogen content of biochar measured by the X-ray photoelectron spectroscopy (XPS), it was proposed that interactions between sulfide and pyridinic nitrogen on biochar surface generate nucleophilic species (e.g., C–S–S) that are highly reactive with chlorinated solvents.^22^ However, different types of N functional groups exist in PCM, which are either formed during PCM production or incorporated afterward for engineering applications.^25−27^ Various types of N functional groups often present in mixtures (e.g., pyridine, pyridinium cations, or quaternary ammonium);^28,29^ however, the identity of N groups responsible for the enhanced PCM reactivity remains unclear. Determining this and understanding the mechanism by which these groups accelerate the reduction of contaminant by sulfide should facilitate the design and production of biochars. Specifically, selected surface functional groups can be populated to increase the reactivity of biochars, thus facilitating the remediation of groundwater contaminated with TCP and other haloalkanes (e.g., 1,2-dichloroethane).

PCM-like polymers (PLPs) are synthetic polymers with properties similar to PCM.^30−32^ Both PLPs and PCM (1) are highly porous with large surface area, (2) are amorphous and conjugated, and (3) have high affinities toward organic contaminants. However, compared with PCM, PLPs are readily synthesized with controlled and homogeneously distributed properties (e.g., type and quantity of functional groups). Previously, PLPs have been used as a framework to prepare model polymer systems for systematic studies of the PCM properties that control surface hydrolysis and reduction reactions.^32−34^ In this study, we employed PLPs containing different nitrogenous functional groups to delineate their contributions to the degradation of TCP by *TOT*HS (total hydrogen sulfide)—the sum of H_2_S, HS^–^, and S^2–^. Specifically, we synthesized four PLPs containing hydroxyl, pyridinic, quaternary ammonium, and pyridinium cation groups, which are abbreviated as PLP-OH, PLP-py, PLP-QA, and PLP-py^+^, respectively. We used TCP as a model contaminant and monitored the kinetics and products of its degradation in the presence of the four PLPs and *TOT*HS at 25 °C. For the two most reactive PLPs, we then measured the kinetics of TCP degradation at various temperatures (i.e., 5–65 °C) and calculated the activation energy (*E*_a_) for the reaction. Furthermore, we examined TCP degradation kinetics in the presence of co-contaminant [i.e., 1,1,1-trichloroethane (TCA) or trichloroethylene (TCE)] and natural organic matter (i.e., Suwannee River natural organic matter, SRNOM).^35,36^ We also proposed the reaction mechanism for the accelerated TCP degradation by PCM. Our work suggests a novel reaction pathway for TCP degradation in a heterogeneous system, provides critical information for the design of reactive adsorbents for TCP destruction, and sheds light on the thermal treatment of chlorinated solvents (e.g., TCP and 1,2-dichloroethane) in groundwater remediation.

## Materials and Methods

### Chemicals

All chemicals were used without further purification, unless otherwise stated. Sigma-Aldrich (Milwaukee, MI): 1,2,3-trichloropropane (TCP), 2,5-dibromohydroquinone, 2,5-dibromopyridine, tetrakis(triphenylphosphine)palladium(0) (>99.5%), copper iodide (CuI, >98%); dimethyformide (DMF, HPLC grade), triethylamine (Et_3_N, >99.5%), chloroform (>99.5%), methanol (>99.9%, HPLC grade), acetone (>99.9%, HPLC grade), acetonitrile (>99.9%, HPLC grade), glycidyltrimethylammonium chloride (GTAC, technical, >90%), iodomethane (CH_3_I, >99%), hydrogen peroxide solution (30%, w/w), and urea (99.0–100.5%, ACS). VWR (Radnor, PA): sulfuric acid (95–98%, ACS grade). Fisher Scientific (Pittsburgh, PA): sodium hydroxide (NaOH, >97.9%). Tokyo Chemical Industry (TCI, Tokyo, Japan): 1,3,5-triethynylbenzene (>98%). A Milli-Q-plus purification system (MilliporeSigma, MO) was used to obtain deionized water (DI water, conductivity ≥18.2 MΩ·cm). Commercial hydroxyl group functionalized multiwalled carbon nanotubes (CNT-OH) were purchased from a verified vendor (Cheap Tubes, Inc., Grafton, VT).

### PLPs Synthesis and Post-Modification

**Scheme 1:**
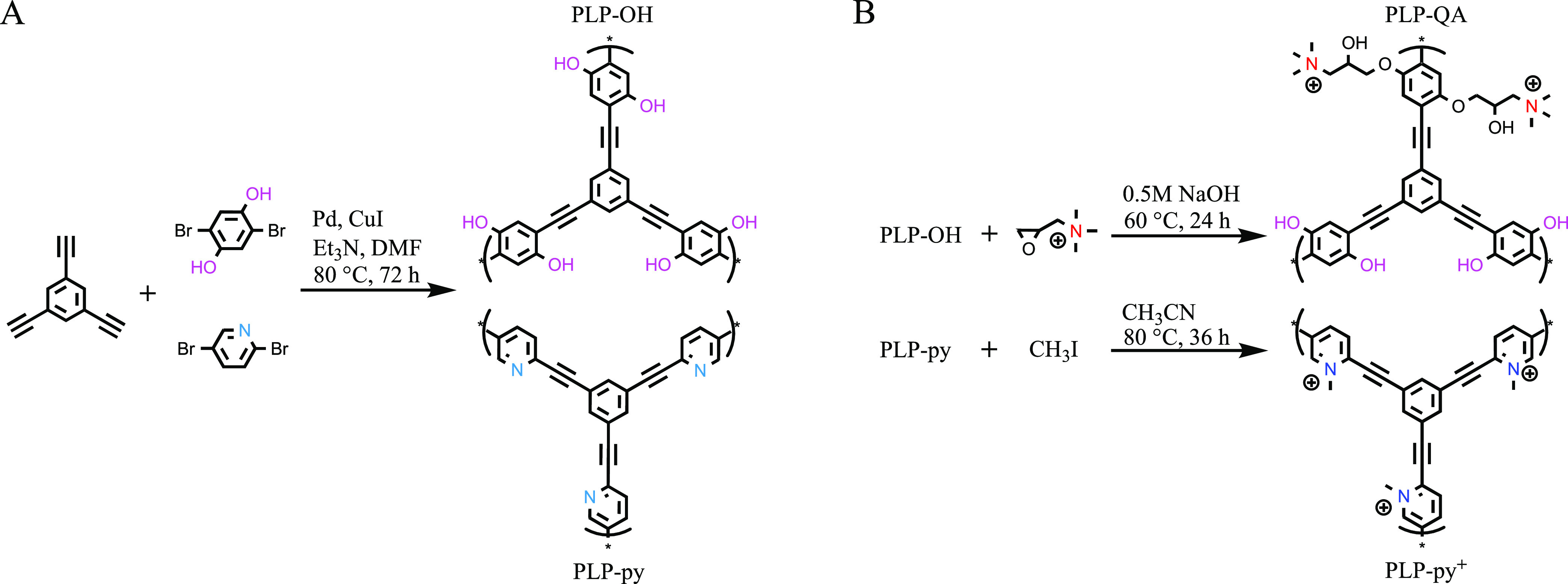
Synthetic Routes for PLPs of (A) Hydroxyl (PLP-OH) and Pyridine (PLP-py) Groups, and (B) Post-Modification for Quaternary Ammonium (PLP-QA) and Pyridinic Cation (PLP-py^+^) Groups

Synthesis of PLP-OH and PLP-py was carried out using a Pd(0)/Cu(I)-catalyzed Sonogashira–Hagihara cross-coupling polymerization method adopted from previous studies as shown in Scheme 1A.^31,37^ Briefly, a mixture of 1,3,5-triethynylbenzene (1 mmol), 2,5-dibromohydroquinone or 2,5-dibromopyridine (1 mmol), tetrakis(triphenylphosphine)palladium(0) (50 mg), and CuI (15 mg) were mixed with DMF (5 mL) and Et_3_N (5 mL), which was then stirred at 50 rpm, 80 °C in darkness for 72 h under N_2_. The product then underwent vacuum filtration with 11 μm Whatman filter paper and was subsequently washed with chloroform, acetone, water, and methanol to remove any residual solvents or unreacted chemicals. Further purification proceeded via Soxhlet extraction using methanol for 48 h. Finally, the solid was vacuum-dried at 60 °C for 24 h and stored in a desiccator before use.

The PLP-QA was obtained by post-modification of PLP-OH following a procedure adopted from a previous study as shown in Scheme 1B (top).^38^ Overall, 0.8 g of PLP-OH was mixed with 8 mL of 0.5 M NaOH and 4.5 g of GTAC (*d* = 1.13 g·cm^–3^) under N_2_ and further reacted at 60 °C in darkness. The surface density of the QA functional groups was modified by adjusting the mass ratio of GTAC relative to PLP-OH (i.e., 5.6, 1.4, and 0.7),^38^ which were abbreviated as PLP-QA_high_, PLP-QA_mid_, and PLP-QA_low_, respectively. The PLP-py^+^ was produced by post-modification of PLP-py following a method adapted from a previous study (Scheme 1B (bottom)).^39^ Briefly, 0.3 g of PLP-py, 36 mL of acetonitrile, and 13.8 g of CH_3_I (*d* = 2.28 g·cm^–3^) were mixed under N_2_ for 36 h at 80 °C in darkness. Similarly, the surface density of the py^+^ functional group was modified by solely adjusting the mass ratio of CH_3_I relative to PLP-py (i.e., 46, 1.9, and 0.95),^40^ which were abbreviated as PLP-py_high_^+^, PLP-py_mid_^+^, and PLP-py_low_^+^, respectively. Samples for Raman spectroscopy were prepared by mixing PLP-py^+^ with *TOT*HS for 5 days at room temperature, followed by rinsing with DI water three times to ensure the removal of *TOT*HS, verified with a colorimetric method,^41^ and air-dried in an anaerobic glovebox (5% H_2_, 20% CO_2_, 75% N_2_, Coy Laboratory Product, Inc., Grass Lake, MI) before use. All PLPs were purified and stored following the same protocol.

### Preparation of PCM-QA and N-Doped Biochar

PCM, namely, CNT and biochar, were grafted with QA and py^+^ functional groups to validate the respective role of these functional groups in mediating TCP degradation in engineering systems. Specifically, commercially available CNT-OH was used as received for QA enrichment following the same protocol for PLP-QA_high_. A biochar made in house was selected as the base material for N-doping. Briefly, the biochar was produced using a switchgrass feedstock (*Panicum vigartum*) at 300 °C under oxygen-limited conditions in a muffle furnace (Model 550-58, Fisher Scientific) for 2 h. The obtained biochar, abbreviated as G300, was ground and passed through a sieve (*d* < 212 μm) to obtain fine powders. To populate the oxygenated function groups, 1 g of G300 biochar was mixed with 10 mL of Piranha solution [3:1 (v/v) mixture of sulfuric acid and hydrogen peroxide] for 1 h at 90 °C. The obtained biochar was abbreviated as OG300 and rinsed with DI water multiple times to remove any residual reagent. For N-doping, we followed a previously published method,^42^ in which 100 mg of OG300 was mixed with 10 g of urea solid in 10 mL of DI water. Subsequently, the mixture was sealed and placed in a 50 mL poly(tetrafluoroethylene) (PTFE)-lined hydrothermal synthesis autoclave reactor (Baoshishan, Henan, China) at 180 °C for 4 h. The obtained N-doped biochar, abbreviated as NOG300, was then washed several times with DI water and dried at 60 °C under vacuum for 24 h before use. NOG300 was further modified following the previous procedures for PLP-py_high_^+^ to obtain pyridinium NOG300 (NOG300-py^+^).

### Batch Experiment Setup

Unless otherwise stated, all aqueous solutions in this study were purged with N_2_ for 2 h and stored in the anaerobic glovebox (O_2_ < 5 ppm). TCP degradation experiments proceeded with the addition of 100 μL TCP (1 g·L^–1^ in MeOH) to borosilicate glass reactors containing preweighed PLP (0.7 g·L^–1^) with 5 mM *TOT*HS in 20 mM phosphate buffer solution (PBS, pH 7). The allyl chloride experiment was conducted with the addition of 100 μL of allyl chloride (1 g·L^–1^ in MeOH) under the same experimental condition. All batch reactors were filled to 14 mL with minimal headspace to eliminate the liquid–gas partitioning of TCP, capped tightly with Teflon-lined septa caps, and wrapped with PTFE tape to prevent potential TCP leakage. The reactors were placed on an end-to-end rotator at 30 rpm in the dark in a Model VRI6P incubator (VWR International, Radnor, PA). Replicate samples were periodically collected for analysis. At the time of sampling, all samples were centrifuged (Eppendorf 5810R, Eppendorf AG, Hamburg, Germany) at 3500 rpm for 5 min to terminate the reaction by separating the solid and aqueous phases. The centrifuge time is included in the reaction time. The aqueous phase was transferred into another vial for the liquid–liquid extraction of TCP, wherein an equal volume of hexane was added to the aqueous sample and vortexed for 3 min. TCP was extracted from the solid phase with 10 mL of hexane/acetone (1:1, v/v) mixture and vortexed for 3 min. All extracts were analyzed by gas chromatography with an electron capture detector (GC-ECD).

### Adsorption Isotherm

The adsorption isotherm of SRNOM on PLPs was carried out in duplicate using a constant solid-to-liquid ratio (0.7 g·L^–1^) at 25 °C and pH 7. Concentrations of SRNOM from 1 to 250 mg_C_·L^–1^ were added to 14 mL batch reactors containing preweighed PLP-QA or PLP-py^+^, capped, and placed on an end-to-end rotator at 30 rpm in the dark. After 5 days, samples were centrifuged and the supernatant was analyzed for the organic carbon content using a total organic carbon (TOC) analyzer. The obtained isotherms for each PLP were fitted using the Freundlich model (*q*_e_ = *K*_f_·C_e_^1/*n*^), where *K*_f_ represents the Freundlich affinity constant, and *n* is the heterogeneity index.^43^

### Material Characterization and Analytical Methods

The infrared spectra were obtained using Fourier transform infrared spectroscopy (FTIR, PerkinElmer 1600 series) with a resolution of 4 cm^–1^ in a range of 4000–750 cm^–1^. Scanning electron microscopy (SEM; Hitachi S-4800, Tokyo, Japan) at an accelerating voltage of 5 kV was used to observe the surface morphologies. The ^13^C solid-state nuclear magnetic resonance (^13^C NMR) was performed using a Bruker Avance 400 spectrometer at 100 MHz ^13^C frequency with 4 mm sample rotors in a double-resonance probe head. The NMR spectra were obtained using ^13^C multiple cross-polarization/magic angle spinning (multi-CP/MAS) NMR technique with a spinning rate of 14 kHz and 90° pulse length of 4 μs for ^13^C. XPS was performed on a PHI 5000 VersaProve with both survey and high-resolution spectra using a 200 μm, 50 W beam with 117 and 23 eV pass energies, respectively. Charge correction was performed on all XPS data to adventitious carbon at a 284.8 eV binding energy. Point-of-zero charge (PZC) of PLPs was measured with a Particle Size and Zeta Potential Analyzer NanoBrook Omni (Brookhaven) using phase analysis light scattering (PALS) mode with default parameters at 25 °C in DI water with a range of adjusted pH conditions. The conductivity was measured using a two-probe bed technique following our established protocol.^33^ Raman spectroscopy was performed on an alpha300R Raman Imaging Microscope (WITec GmbH, Germany) with a 532 nm laser and 100s integration time for each data point. The nonpurgeable organic carbon (NPOC) of the SRNOM was determined with a TOC analyzer (TOC-L, Shimadzu, Japan). A GC-ECD (Agilent 6890N, Santa Clara, CA) equipped with a Rxi-5 ms column (30 m 0.25 mm i.d., Restek, Bellefonte, PA) was used for quantitative analysis of TCP following EPA method 551.1.^44^ Chloride was analyzed with a Shodex SI-52 4E anion column (Showa Denko, Tokyo, Japan) using ion chromatography (IC) coupled with a conductivity detector on an HPLC system (Shimadzu, Kyoto, Japan). The mobile phase was 3.6 mM Na_2_CO_3_ buffer, the flow rate was 0.9 mL·min^–1^, and the oven temperature was 45 °C. The data analysis was performed using Igor Pro (Wavemetrics, Lake Oswego, OR), and all of the reported data were derived from triplicate samples with 95% confidence level.

## Results and Discussion

### Surface Characterization of PLPs

The PLPs were first characterized by FTIR spectroscopy. The disappearance of terminal alkyne signal (Figure S1; 3280 cm^–1^, –C≡C-H) in 1,3,5-triethynylbenzene, and the appearance of quaternary alkyne signal (2200 cm^–1^, R-C≡C-R′) in all of the PLPs indicate the successful cross-coupling reaction between aryl halide and terminal alkyne, which are in line with previous studies.^33,34,45−47^ Furthermore, the successful incorporation of –CH_3_ at 2924 and 3000 cm^–1^ for PLP-QA and PLP-py^+^ confirmed the formation of QA and py^+^ on the PLP surface.

**Figure 1:**
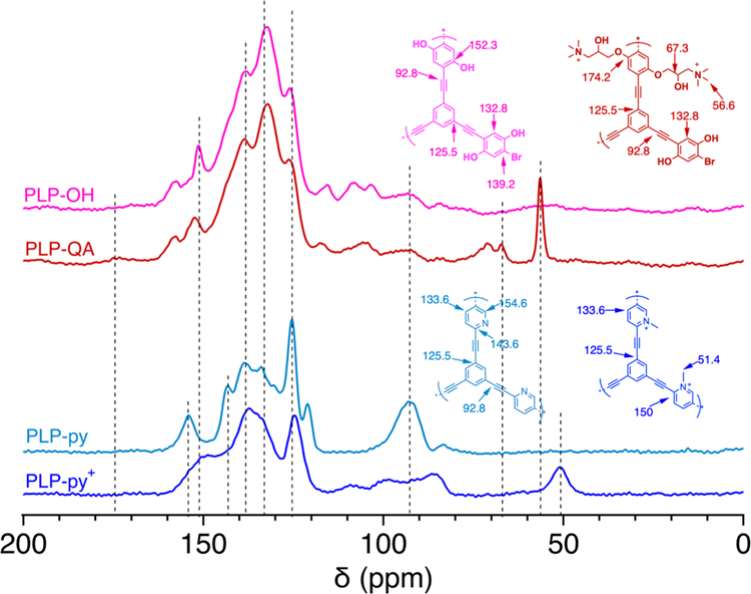
Solid-state ^13^C multi-CP/MAS NMR and peak assignments of PLP-OH (pink), PLP-QA (red), PLP-py (cyan), and PLP-py^+^ (blue) in this study. All spectra were recorded at a spinning speed of 14 kHz with a 90° ^13^C pulse length of 4 μs.

Further characterization was performed by ^13^C solid-state NMR to gain a better understanding of the PLP structures at the molecular level (Figure 1). Overall, consistent with our previous work, the chemical shifts at 125.5 ppm (C_Ar_-C≡C–C_Ar_), 132.8 ppm (C_Ar–H_), and 92.8 ppm (C_R-C≡C-R′_) suggest the formation of conjugated polymer networks in synthesized PLPs.^33,34^ In PLP-OH (pink), the chemical shift at 152.3 ppm was assigned to the aromatic carbon that connects to the hydroxyl group (C_Ar–OH_).^48,49^ After quaternization with epoxide derivatives, PLP-QA (red) retained all peaks of PLP-OH while exhibiting additional peaks at 56.6 ppm (methyl carbon), 67.3 ppm (aliphatic hydroxyl carbon, C_–OH_) and 174.2 ppm (aromatic carbon with ether group, C_Ar–O-R_)^34^ due to the successful incorporation of QA groups. The co-occurrence of both –OH and methyl groups in PLP-QA (red) indicates that some –OH groups on the PLP surface were successfully grafted with QA, as shown in Scheme 1B. Two signature chemical shifts of pyridinic carbon at 143.6 and 154.6 ppm were observed with the polymer backbone of PLP-py (cyan),^50,51^ which diminished in PLP-py^+^ (blue) after post-modification.^52^ Moreover, the occurrence of resonance at 51.4 ppm (methyl carbon) confirmed the existence of the pyridinium cation on PLP-py^+^. Detailed assignments on the chemical shift of NMR spectra for all PLPs are provided in Table S1.

The ζ-potentials and PZC values of all four PLPs were also characterized. The ζ-potential for all PLPs decreased as pH increased from 2 to 10 (Figure S2A). The measured PZC (defined as the point of pH at which the net surface charge equals zero)^53^ of PLP-OH, PLP-py, PLP-py^+^, and PLP-QA were 3.2, 3.5, 7.8, and 8.6, respectively, suggesting that the incorporation of QA and py^+^ functionalities clearly made the PZC values of these PLPs more positive. We also measured the ζ-potentials of PLP-py^+^ and PLP-QA with varying surface charge densities. As shown in Figure S2B, the ζ-potentials of PLP-py^+^ and PLP-QA demonstrated an increasing trend at pH 7 from low, medium, to high charge densities. The surface functional group contents of QA and py^+^ were subsequently quantified by XPS (Figures S3–S5). We found that the total nitrogen content increased to 1.51, 1.87, and 2.44% for PLP-QA_low,_ PLP-QA_mid,_, and PLP-QA_high_, respectively (Figure S3A–C). This is supported by our results from the peak deconvolution of C 1s (Figure S4A–C) and N 1s spectra (Figure S5A–C), where the percentage of C–N^+^ increased from 12.52, 14.27, to 19.11% in the C 1s spectra and from 74.14, 76.70, to 84.65% for C–N^+^ in the N 1s spectra for PLP-QA_low_, PLP-QA_mid_, and PLP-QA_high_, respectively.^54,55^ Although the total nitrogen content for PLP-py_low_^+^, PLP-py_mid_^+^, and PLP-py_high_^+^ remained unchanged, our results from the peak deconvolution of N 1s spectra (Figure S5D–F) suggest an increase in C–N^+^ of 44.85, 47.91, and 64.96% for PLP-py_low_^+^, PLP-py_mid_^+^, and PLP-py_high_^+^, respectively.^56,57^ Overall, our XPS results confirmed that the surface densities of functional groups were successfully tuned from low, medium, to high for both PLP-QA and PLP-py^+^.

### TCP Degradation in the Presence of PLPs and *TOT*HS

**Figure 2:**
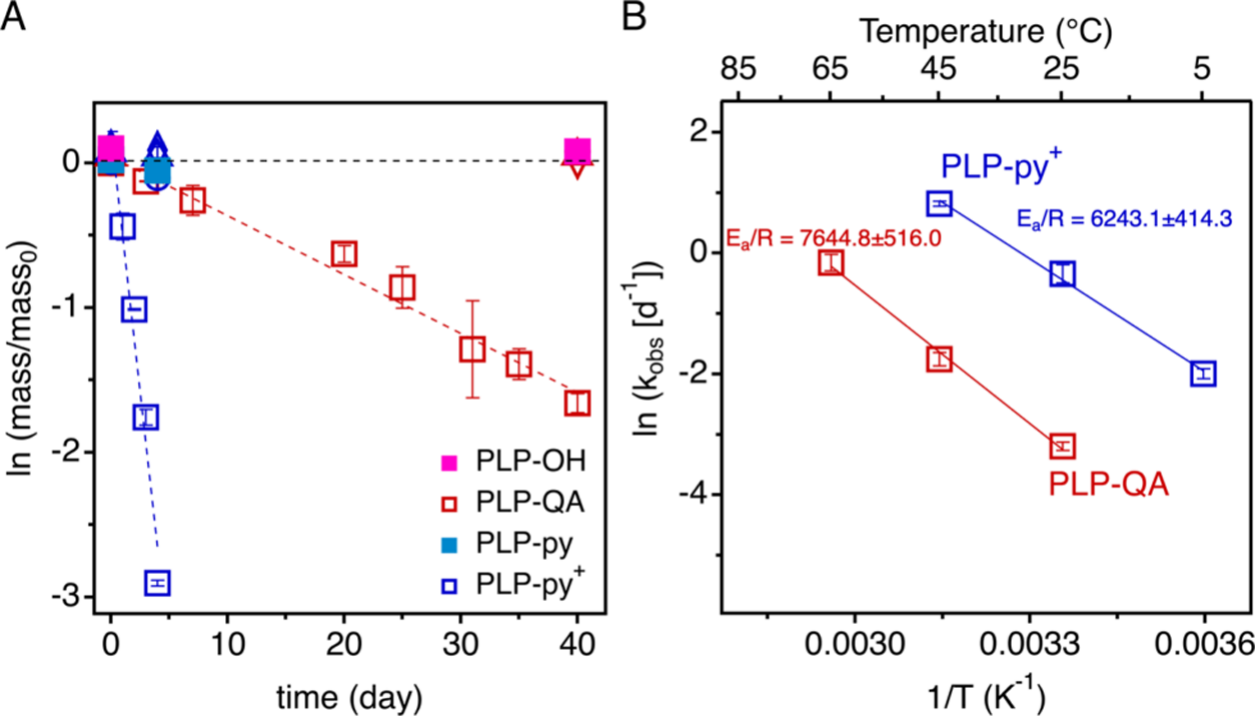
(A) First-order kinetic reaction of 1,2,3-trichloropropane (TCP) degradation in the presence of 5 mM *TOT*HS at pH 7 (20 mM phosphate buffer) and 0.7 g·L^–1^ of quaternary ammonium (QA) grafted PCM-like polymer (PLP-QA, red hollow squares), pyridinium cation (py^+^) grafted PLP (PLP-py^+^, blue hollow squares), hydroxyl group (–OH) grafted PLP (PLP-OH, red solid squares), and pyridine (py) grafted PLP (PLP-py, blue solid squares) under room temperature (25 °C). Dashed lines represent the best fit of experimental data points of PLP-QA (red) and PLP-py^+^ (blue) with r-squared (*r*^2^) of 0.983 and 0.967, respectively. The observed reaction rate (*k*_obs_) of PLP-QA and PLP-py^+^ was 0.041 ± 0.002 and 0.717 ± 0.077 d^–1^, respectively. Experimental controls include TCP without *TOT*HS (circles), PLP (triangles), or both (diamond). The error bars were derived from triplicates. (B) Arrhenius plot (*k*_obs_ = *A* e^–*E*_a_/*RT*^) of 1,2,3-trichloropropane (TCP) degradation for PLP-QA (red) and PLP-py^+^ (blue). The solid lines indicate the best fit of data to the Arrhenius equation. The calculated activation energy of TCP degradation in the presence of PLP-QA (red) and PLP-py^+^ (blue) are 63.6 ± 6.1 and 51.9 ± 4.9 kJ·mol^–1^, respectively. The error bars were derived from triplicate samples with a 95% confidence level.

The transformation of TCP was first examined in the presence of the four synthesized PLPs (namely, PLP-OH, PLP-QA PLP-py, and PLP-py^+^) in 5 mM *TOT*HS at 25 °C (Figure 2A). Only PLP-QA and PLP-py^+^ gave significant degradation of TCP, whereas no change in TCP concentration was observed in samples containing PLP-OH or PLP-py with *TOT*HS. Controls containing TCP and *TOT*HS in the absence of PLP also showed no degradation. Additional experiments (Figure S6) showed negligible TCP degradation in the presence of trace palladium catalyst residue^58,59^ and post-modification reagents (i.e., GTAC or CH_3_I), further confirming that the accelerated TCP degradation was due to the PCM-sulfide synergy. We calculated the enthalpy and entropy changes (i.e., Δ*H* and Δ*S*) of TCP on PLP-py^+^, which were −10.0 and 82.8 kJ·mol^–1^, respectively. It suggests that the adsorption of TCP onto PLPs is an exothermic process (Δ*H* < 0) with increasing disorder of the system (Δ*S* > 0). However, we believe the impact of surface adsorption on the reaction kinetics is limited because most TCP is associated with the surface to begin with. Throughout the sampling points, the amount of TCP in the aqueous phase was negligible (<10%). The observed first-order reaction rate (*k*_obs_) of TCP degradation with PLP-QA and PLP-py^+^ are 0.041 ± 0.002 d^–1^ and 0.717 ± 0.077 d^–1^, respectively, corresponding to half-lives (*t*_1/2_) of 16.91 ± 1.17 and 0.98 ± 0.15 days (Table S2). During these experiments, chloride (Cl^–^) was formed at a molar ratio of nearly 3:1 (formed Cl^–^ vs degraded TCP; Figure S7) for both PLP-QA and PLP-py^+^.

To understand the role of PCM in facilitating TCP degradation, we determined the rate constants of TCP degradation in the presence of PLP-QA and PLP-py^+^ at three different temperatures. Three temperatures (i.e., 25, 45, and 65 °C) were selected for the PLP-QA system, whereas lower temperatures (i.e., 5, 25, and 45 °C) were chosen for the PLP-py^+^ system due to its faster kinetics compared to those of the QA system. TCP degradation exhibited pseudo-first-order kinetics, as shown in Figure S8. The observed rate constants (*k*_obs_) of TCP degradation in the presence of PLP-QA increased from 0.041 ± 0.002 to 0.86 ± 0.08 d^–1^ from 25 to 65 °C (Figure S8A–C), corresponding to a decrease in half-lives (*t*_1/2_) from 16.91 ± 1.17 to 0.81 ± 0.11 days, respectively. TCP degradation by *TOT*HS in PLP-py^+^ (Figure S8D–F) exhibited faster kinetics. The observed rate constants (*k*_obs_) for TCP degradation increased from 0.14 ± 0.01 to 2.27 ± 0.06 d^–1^ from 5 to 45 °C, corresponding to half-lives of 5.13 ± 0.41 and 0.31 ± 0.01 d^–1^, respectively. All values are summarized in Table S2.

In Figure 2B, the apparent activation energies (*E*_a_) of TCP degradation were 63.6 ± 6.1 and 51.9 ± 4.9 kJ·mol^–1^ for PLP-QA and PLP-py^+^, respectively. Statistical analysis (*t*-test, *p* < 0.05) suggests that the difference between the obtained *E*_a_ values from two PLPs is significant, which indicates the chemical identity of N functional groups plays a key role in TCP activation. Previous studies reported that N-doping can increase the reactivity of PCM toward the dechlorination of TCE and PCE,^22,24^ and the pyridinic group was proposed to be responsible. Other studies proposed that the oxygenated functional groups, namely, –OH, can act as a strong base and thus accelerate the dechlorination of 1,1,2,2-tetrachloroethane or hexachloroethane by *TOT*HS.^60,61^ Our study, for the first time, clearly demonstrates that the N functional group identity is critical. For instance, some N functional groups can be highly reactive (i.e., QA and py^+^), whereas others can be inactive (i.e., py). Moreover, the presence of py^+^ functional groups accelerated TCP degradation rates by 13.1- to 17.5-fold compared to QA under the same experimental conditions. The difference in the reactivities of PLP-QA and PLP-py^+^ could be attributed to several factors. First, the total N content of PLP-py^+^ (i.e., 4.77%) was approximately twice as high as that of PLP-QA (i.e., 2.44%) according to the XPS survey results (Figure S3). Moreover, py^+^ groups appear to be more effective in activating TCP and thus further reduce the *E*_a_ value of the reaction by 18.4% compared to that of QA groups. Cross comparison (Figure S9) to other studies suggests that the mass-normalized rate constants (*k*_M_) for TCP in the presence of PLP-QA and PLP-py^+^ are much faster than those reported for zerovalent iron/zinc (ZVI/ZVZ) system (i.e., *k*_M_ = 2.44 × 10^–3^ L·g^–1^·h^–1^ for PLP-QA vs 4.27 × 10^–2^ L·g^–1^·h^–1^ for PLP-py^+^ vs 0.01–1.9 × 10^–3^ L·g^–1^·h^–1^ for ZVI/ZVZ).^1,2,62^

### Influence of Functional Group Density, Co-contaminant, and SRNOM

**Figure 3:**
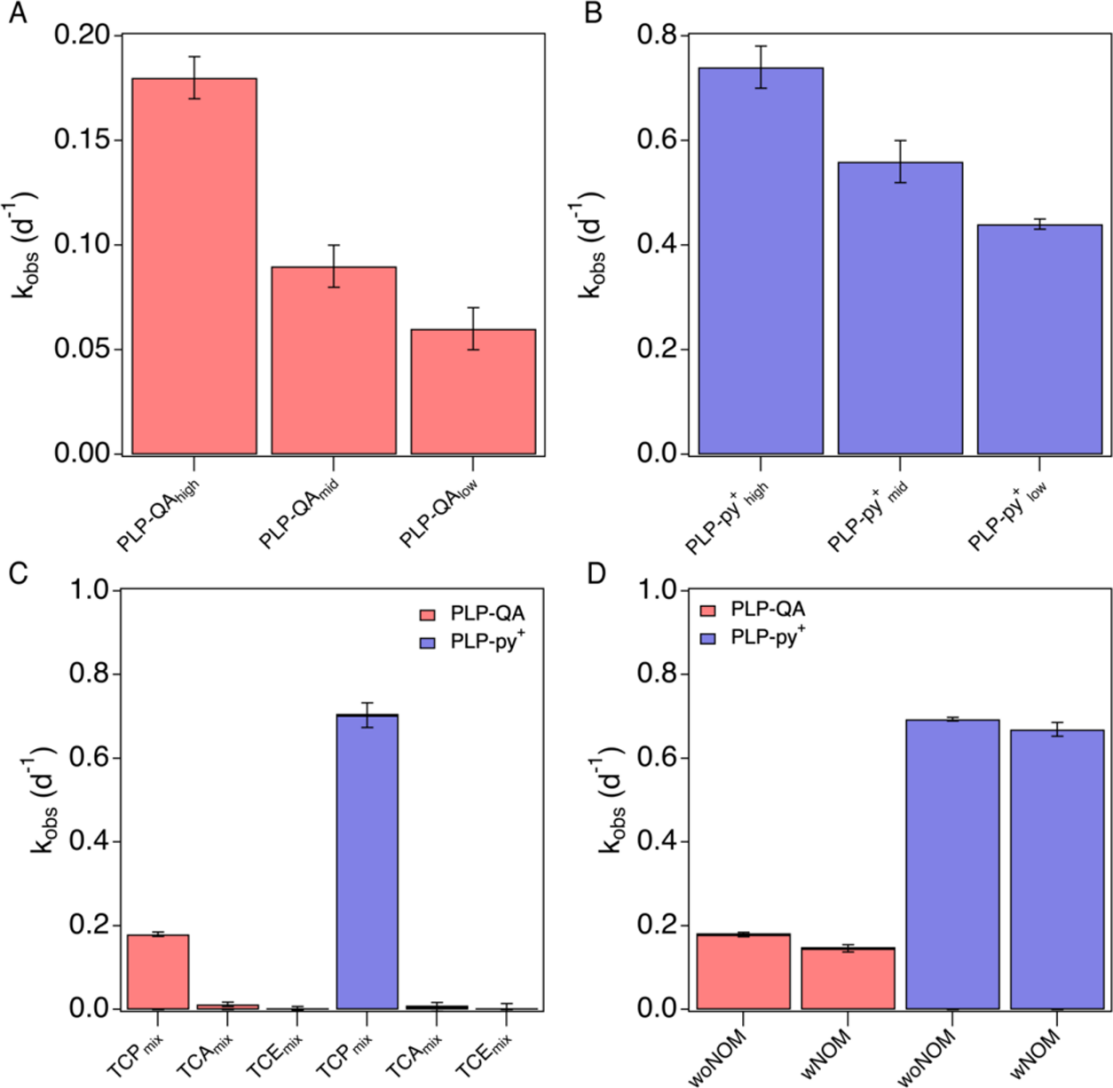
1,2,3-Trichloropropane (TCP) degradation in the presence of 5 mM *TOT*HS in 20 mM phosphate buffer (PBS, pH 7) and 0.7 g·L^–1^ of (A) PLP-QA of different surface QA density at 45 °C, (B) PLP-py^+^ of different density of surface pyridinium cation at 25 °C, (C) PLP-QA and PLP-py^+^ co-existing with TCA, TCE, and (D) PLP-QA and PLP-py^+^ co-existing with NOM. The labels of TCA_mix_, TCE_mix_, and TCP_mix_ indicate that contaminants were introduced to the reaction system as a mixture of three, while woNOM or wNOM indicates the absence or presence of NOM in the reaction system. The error bars were derived from triplicate samples with a 95% confidence level.

To explore how surface group densities can affect TCP degradation, we modified PLP-QA and PLP-py^+^ to obtain three different surface coverages (i.e., PLP-QA_low_, -QA_mid_, -QA_high_, -py_low_^+^, -py_mid_^+^, and -py_high_^+^), which was confirmed by their PZC and XPS measurements (Figures S2B and S3–S5). As shown in Figure 3A, the rate constants for TCP degradation by PLP-QA decreased as the surface group density decreased. The observed rate constant for PLP-QA_high_ was the highest (*k*_obs_ = 0.18 ± 0.01 d^–1^), followed by PLP-QA_mid_ (*k*_obs_ = 0.09 ± 0.01), and PLP-QA_low_ (*k*_obs_ = 0.06 ± 0.01 d^–1^). A similar trend was observed for PLP-py^+^ (Figure 3B), where the *k*_obs_ values were 0.74 ± 0.04, 0.56 ± 0.04, and 0.44 ± 0.01 d^–1^ for PLP-py_high_^+^, PLP-py_mid_^+^, and PLP-py_low_^+^, respectively.

We then examined the influence of the co-contaminants (i.e., TCA and TCE) and SRNOM on TCP degradation. We selected TCA and TCE in this study partially due to their co-occurrence with TCP in the environment. But more importantly, they are included because they are structural analogs to TCP, which allows us to verify our proposed reaction mechanism for TCP.^63,64^ SRNOM was included as the source of organic carbon to reflect the environmental conditions. The result was summarized in Figure 3C,D. Experiments were conducted at 45 °C for PLP-QA, but at 25 °C for PLP-py^+^ to allow for comparable monitoring times. Remarkably, both TCA and TCE degradations were negligible in the presence of PLP-QA or PLP-py^+^, whereas the *k*_obs_ for TCP degradation was similar to that without the addition of co-contaminants. Presumably, the differences in reactivity of TCA, TCE, and TCP are due to differences in their structural and/or reaction pathways, which are discussed in the next section. The addition of 3 mg·L^–1^ of SRNOM decreased the *k*_obs_ values for TCP by 18.4 and 3.5% for reactors containing PLP-QA and PLP-py^+^, respectively, whereas no TCP decay was found without PLP. Our results from the adsorption isotherm (Figure S10) showed higher values of *K*_f_ for PLP-QA compared to PLP-py^+^ (Table S3), suggesting that PLP-QA has higher adsorption affinity toward SRNOM than PLP-py^+^.^43,65^ This could be attributed to the lower content of N functional groups incorporated in PLP-QA (Figure S3), making the surface less hydrophilic and thus accumulating more organic matter. Similar observations were found in previous studies on polymer films.^66,67^ The larger decrease in TCP decay rates in the presence of PLP-QA in comparison to PLP-py^+^ can be explained by more SRNOM blocking the surface reactive sites of PLP-QA.

### Reaction Mechanism

Multiple reaction pathways could be responsible for TCP dechlorination in the PCM-sulfide system: (1) hydrogenolysis that yields 1,2- or 1,3-dichloropropane, 1- or 2-chloropropane, or propane, (2) hydrolysis that results in the formation of 1,3- or 2,3-dichloro-1-propanol, (3) elimination that produces 3-chloro-1-propene (reductive), 1,3- or 2,3-dichloro-1-propene (nonreductive), or (4) nucleophilic substitution that generates S-adducts as products.^17^ To facilitate the detection of these possible reaction products, a separate experiment was performed starting with a 10-fold higher TCP concentration (i.e., 480 μM). In this experiment, neither dichloropropane nor monochloropropane was observed in either aqueous or solid phase extracts using GC-MS, which eliminates the possibility that hydrogenolysis was significant. Hydrolysis was unlikely, because the experiments were conducted at pH 7, and none of the putative hydrolysis products were detected. It is also unlikely the conductivity of PLPs and PCMs played an important role in facilitating TCP decay, given that none of the PCMs without N modification were reactive (see next section) despite being conductive (Table S4).^68^ Furthermore, all four PLPs are nonconductive, but only two of them (i.e., PLP-QA and PLP-py^+^) were able to accelerate TCP decay by sulfide. This further supports our hypothesis that surface functional group identity rather than the conductivity of the material is crucial in promoting TCP degradation. Nucleophilic substitution by (poly)sulfides in the aqueous phase was also insignificant based on control experiments where TCP was exposed to supernatants obtained after reacting PLP-py^+^ and *TOT*HS. Specifically, <10% TCP degradation and no products were observed after 14 days in the supernatant, compared to 80% TCP degradation by *TOT*HS in the presence of PLP-py^+^ after 3 days. Also, the involvement of surface-bound nucleophiles alone^69^ is unlikely due to the lack of reactivity when TCP was exposed to the solid phase of *TOT*HS-pretreated PLP-py^+^. Thus, the simultaneous presence of PLPs and aqueous *TOT*HS seems to be necessary for a significant reduction of TCP.

To further characterize the reaction pathway, we monitored the inorganic product of TCP degradation (i.e., chloride) by IC. We observed mass balance, i.e., complete dechlorination, for TCP degradation by *TOT*HS in batch reactors containing PLP-QA or PLP-py^+^. Specifically, 1.86 ± 0.03 μM and 1.76 ± 0.00 μM chloride (Cl^–^) was formed when 0.66 ± 0.01 μM and 0.61 ± 0.01 μM TCP was transformed by *TOT*HS in the presence of PLP-QA and PLP-py^+^, respectively. The molar ratio yield of chloride (as Δ[Cl^–^]/Δ[TCP]) was 2.8 and 2.9 for PLP-QA and PLP-py^+^, respectively. The mass balance on total chlorine, calculated as the ratio of (3[TCP] + [Cl^–^])/(3[TCP]_0_), was 99.7 ± 5.1% for PLP-QA and 94.5 ± 4.7% for PLP-py^+^, indicating a nearly complete displacement of chlorine. In contrast, no Cl^–^ was observed from TCP in the presence or absence of *TOT*HS with or without PLP-OH and PLP-py.

**Figure 4:**
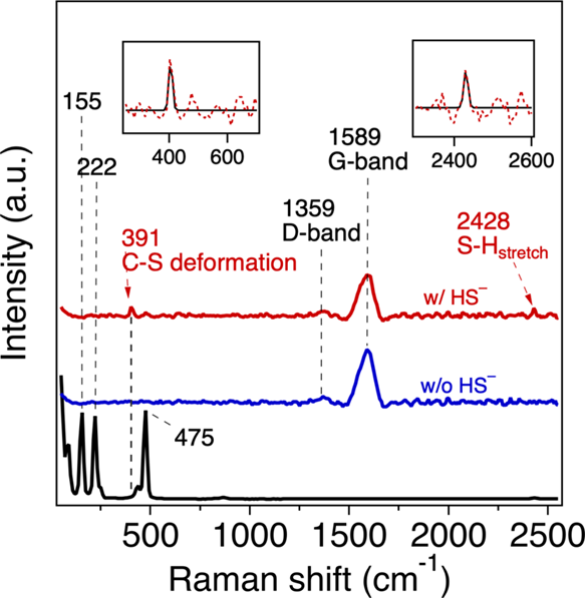
Background subtracted Raman spectrum of PLP-py^+^ after 5 days reaction within the sample group (red) and the control group (blue). Elemental S (black) is also scanned for comparison. Raman condition: wavelength 532 nm laser, integration time 100s. The inserted windows showed the comparison between the scanned spectrum (red dash line) and the fitted peak (black solid line) using pseudo-Voigt function, which is a linear combination of Gaussian function and Lorentzian function.

To further examine the possible activated nucleophilic species containing S on the PLP surface, we performed Raman spectroscopy with PLP-py^+^ exposed to 5 mM *TOT*HS for 5 days. For this experiment, PLP-py^+^ was selected due to its faster reaction rate (see Figure 2A). As shown in Figure 4, there were three signature shifts from elemental sulfur (black: 155, 222, 475 cm^–1^), which were not observed in PLP-py^+^ solid with (i.e., red) or without (i.e., blue) being exposed to *TOT*HS. Compared to the PLP-py^+^ solid (i.e., blue), two additional peaks at 391 and 2428 cm^–1^ showed up in the same sample when exposed to *TOT*HS (i.e., red), which could be assigned to the C–S deformation^70,71^ and S–H stretching,^72^ respectively. Our results support the formation of surface C–S bonds on the PLP surface, and these surface species might serve as nucleophiles in the reaction systems containing both *TOT*HS and PLP-py^+^. Analogous results were reported in previous studies where XPS and X-ray absorption near edge spectroscopy (XANES) were employed to confirm the formation of surface C–S–S species after N-doped biochar was exposed to *TOT*HS.^22,24^

**Figure 5:**
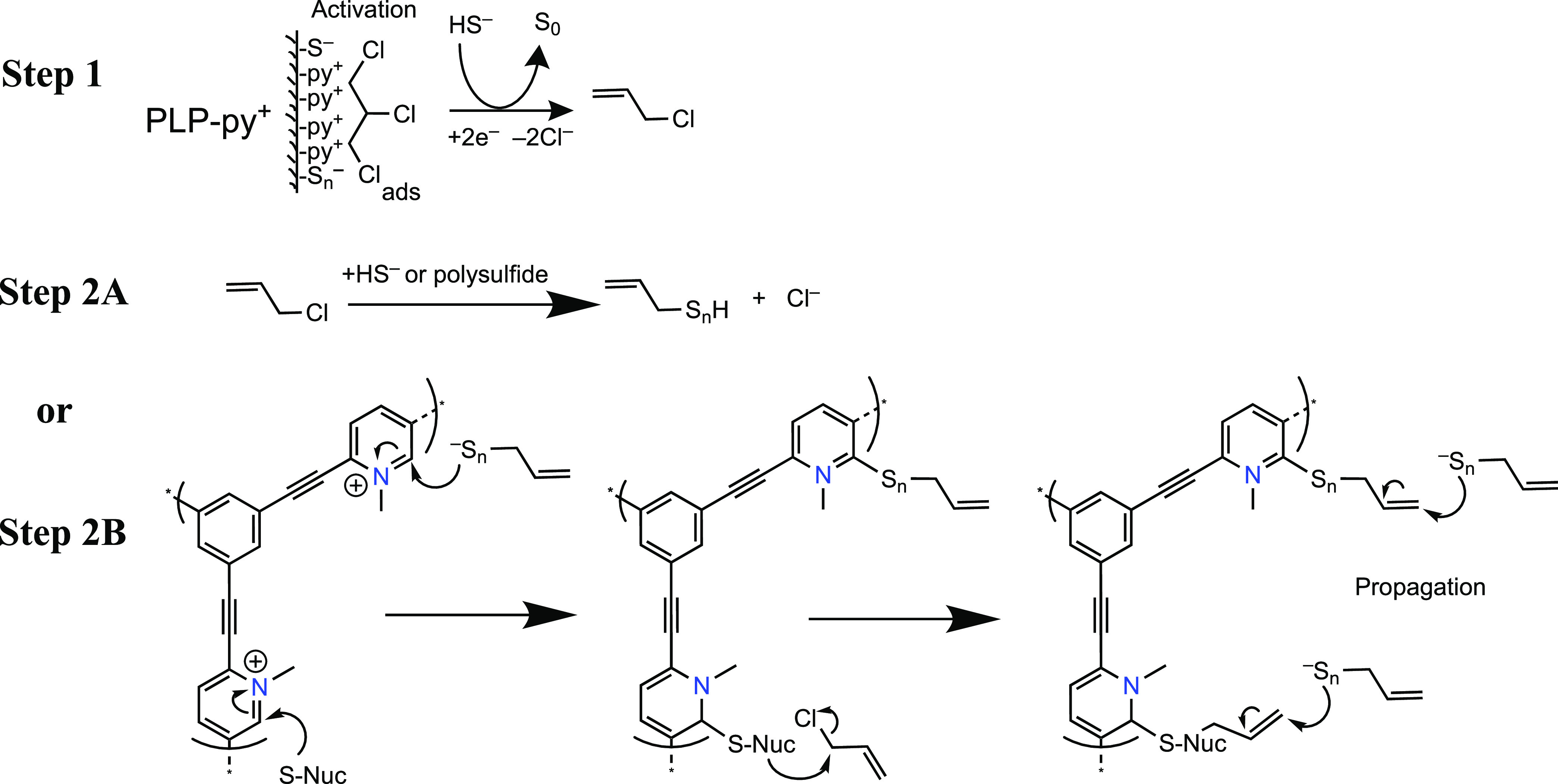
Possible reaction mechanisms for TCP dechlorination in the presence of *TOT*HS and PLP-py^+^. “S-Nuc” represents sulfur nucleophiles such as *TOT*HS and polysulfide. The Michael addition reactants in Step 2B (i.e., C_3_H_5_S*_n_*^–^) are generated from Step 2A, where *n* ≥ 1. Note that PLP-py^+^ is one example of PLPs that undergo the proposed reaction, which also applies to other PLPs and PCM such as PLP-QA, CNT-QA, NOG300, and NOG300-py^+^.

Based on these results, we propose a two-step reaction pathway for PLP-enhanced TCP degradation by *TOT*HS (Figure 5). In the first step, we propose that TCP is activated by PLP-py^+^ and accepts 2 electrons from HS^–^, resulting in the elimination of two chlorines (i.e., reductive ß-elimination) and the formation of allyl chloride as an intermediate product. This type of reaction has been shown to be the most thermodynamically favorable in computational studies,^17,73^ which is also confirmed in experimental studies with chlorinated and brominated solvents containing the vicinal dihalide structure, such as 1,1,1,2- or 1,1,2,2-tetrachloroethane,^74^ hexachloroethane,^75^ and 2,3-dibromopentane.^76^ Conversely, the absence of TCA degradation is consistent with the lack of vicinal dihalide structure in TCA. Also, the lack of TCE degradation may be attributed to its preference to undergo the reductive ß-elimination via a π-bonded trichlorovinyl surface complex through sequential dissociative single electron transfers (SET), as has been shown with zerovalent iron.^77−80^ In contrast, *TOT*HS is not primarily a SET reductant, so the above-mentioned mechanism for TCE is less favorable.^81^ In fact, a previous study reported that the addition of cysteine into a system containing iron sulfide (FeS) significantly slowed the dechlorination of TCE,^78^ and concluded that cysteine did not act as an additional reductant, which is consistent with our hypothesis that sulfur species (e.g., *TOT*HS) do not act as SET reductants in our system.

The formed allyl chloride from the reductive ß-elimination of TCP (Figure 5, step 1) is highly susceptible to hydrogenolysis,^17^ and might also react rapidly with S-nucleophiles.^20,22^ This is supported by our results that faster degradation of allyl chloride was observed with *TOT*HS in the absence of PLP (i.e., *t*_1/2_ = 8.7 ± 0.6 h) than with PLP-py^+^ (i.e., *t*_1/2_ = 23.5 ± 3.6 h) (Figure S11). In the meantime, surface sulfur nucleophiles are likely formed by the reaction between an aqueous nucleophile (e.g., *TOT*HS or polysulfide) and the partially positively charged C adjacent to the N in py^+^ functional group (Figure 5, step 2B), which may further react with allyl chloride for complete dechlorination.^22,24^ The sustained reactivity toward TCP decay in the PLP system can be explained by the potential formation of sulfur adducts, which may form between sulfur nucleophiles and the unsaturated C=C bonds via Michael addition, as illustrated in the last step of Step 2B.^82,83^ The lack of intermediate product detection in our PLP system and the new peak formation during the allyl chloride reaction with TOTHS alone (Figure S12) further suggest that such sulfur adducts are not extractable. Meanwhile, the product from Step 2A (i.e., –CS*_n_*^–^) may also be sequestered by PLPs via the Michael addition to the unsaturated C=C bonds. By contrast, we attribute the lack of reactivity of PLP-py to the less electron-withdrawing effect of pyridinic N, resulting in the lack of surface nucleophile formation in PLP-py system. This is supported by the much larger Hammett constant for N in py^+^ compared with N in pyridine (σ_ortho_ = 3.11 and 0.71, respectively^84^).

### TCP Degradation with Modified PCM

To assess whether the results obtained with PLPs can inform the design of PCM materials, we produced three N-modified PCM: quaternary ammonium groups (CNT-QA) and N-doped biochar (NOG300 and NOG300-py^+^), to test their performance in facilitating TCP degradation.

**Figure 6:**
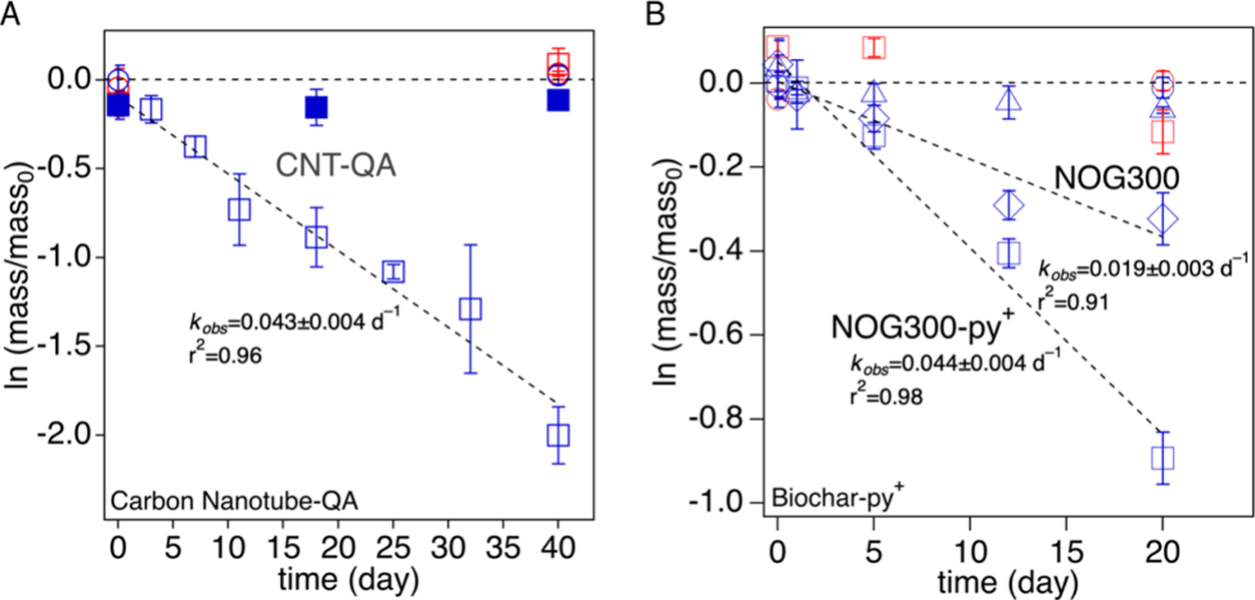
(A) First-order kinetic reaction of 1,2,3-trichloropropane (TCP) degradation in the presence of 5 mM *TOT*HS and 0.7 g·L^–1^ CNT-QA (blue hollow squares) at pH 7 (20 mM phosphate buffer solution, PBS) under 45 °C. The control samples include CNT-OH with (blue solid squares) or without *TOT*HS (red hollow squares), *TOT*HS + TCP (blue hollow circles), and TCP only (red hollow circles). Dashed lines represent the best fit of experimental data points of CNT-QA with r-squared (*r*^2^) of 0.96. The observed reaction rate (*k*_obs_) of CNT-QA was 0.04 ± 0.00 d^–1^. (B) First-order kinetic reaction of TCP degradation in the presence of 5 mM *TOT*HS and 0.7 g·L^–1^ NOG300-py^+^ (blue hollow squares) at pH 7 under 45 °C. The control samples include NOG300-py^+^ + TCP (red hollow squares), *TOT*HS + TCP (blue hollow circles), and TCP only (red hollow circles). The biochar before N dope (i.e., OG300, blue hollow triangles), and the N-doped biochar without methylation (i.e., NOG300, blue hollow diamonds) were also included for comparison. Dashed lines represent the best fit of experimental data points of NOG300-py^+^ and NOG300 with r-squared (*r*^2^) of 0.98 and 0.91, respectively. The *k*_obs_ of NOG300-py^+^ and NOG300 were 0.04 ± 0.00 and 0.02 ± 0.00 d^–1^, respectively. The error bars were derived from triplicate samples with a 95% confidence level.

As shown in Figure 6, accelerated TCP degradation was observed in all three N-modified PCMs, namely, CNT-QA, NOG300, and NOG300-py^+^. In contrast, negligible TCP degradation was observed with PCM containing no or minimum N functional groups (i.e., G300, OG300, and CNT-OH). Pseudo-first-order degradation kinetics were observed for TCP. Specifically, the observed rate constants were 0.043 ± 0.004, 0.044 ± 0.004, and 0.019 ± 0.003 d^–1^ for systems containing CNT-QA, NOG300-py^+^, and NOG300, respectively, corresponding to TCP half-lives of 16.3 ± 1.1 days, 15.9 ± 1.0 days, and 37.4 ± 4.2 days. It is worth noting that the NOG300 also showed reactivity toward TCP, but the reaction rate was more than doubled upon quaternization. These observations further support the reaction mechanism proposed above, where the synergy between *TOT*HS and N functional groups of PCM significantly accelerates TCP degradation, and their reactivity is strongly dependent on the N identity. It also indicates that the microporosity of these materials was not a crucial factor in facilitating TCP decay. Our previous study found that PLP-OH and PLP-QA had comparable micropore volume (0.075 vs 0.062 cm^3^·g^–1^) and total pore volume (0.401 vs 0.434 cm^3^·g^–1^),^34^ while only the latter showed TCP degradation in this study.

### Environmental Significance

Our results have demonstrated the superior reactivity of QA and py^+^ functional groups toward TCP by lowering the *E*_a_ values of the reaction. A novel two-step process for TCP transformation was proposed, resulting in complete dechlorination of TCP. The acquired knowledge herein can be applied to tailor PCM, such as biochar and activated carbon, which have been widely used for contaminant removal. For instance, using the U.S. EPA Work Breakdown Structure model, the cost of TCP treatment to meet the MCL of California (i.e., <5 ng·L^–1^) was estimated to be $0.28/1000 gallons of water produced.^19^ This price corresponds to a total annual cost of $0.75 million or $33.16 million for small (≤1 MGD) and large (>1 MGD) water systems, respectively, which was cost-prohibitive due to the high cost associated with adsorbent regeneration and disposal.^18^ By contrast, our result from this study demonstrated, for the first time, the feasibility of simultaneous adsorption and degradation of TCP using N-modified PCM, which could provide important guidelines for the design of reactive adsorbents and eliminate the need for adsorbent regeneration. For instance, the TCP degradation with NOG300-py^+^was more than 2 orders of magnitude faster than other technologies, such as ZVZ/ZVI (Figure S9), based on the mass-normalized rate constants, signifying the promise of the PCM-sulfide synergy in promoting TCP remediation regarding both economy and efficacy.

Moreover, the thermal treatment of soils contaminated with chlorinated solvents typically requires high temperatures (e.g., 100 °C or higher) and long contact times (e.g., up to 177 days) to achieve sufficient removal, which is not cost-effective.^85,86^ Our results from this study demonstrated that N-modified PCM could not only capture TCP from water but also render the adsorbed TCP susceptible to fast degradation at temperatures (e.g., 45–60 °C) that are much lower than those typically used in the industry. These findings may shed light on innovative thermal treatments for contaminants beyond TCP. For instance, an innovative treatment could incorporate N-modified PCM in a packed bed, which could help concentrate TCP and facilitate their thermal degradation in the subsequent step. Lastly, we proposed a novel reaction pathway for TCP decay. A better understanding of the reaction mechanism could help more accurately predict the transformation products for TCP and other chlorinated solvents with similar structures (i.e., aliphatic hydrocarbon with vicinal dihalogens), such as 1,2-dichloropropane or 1,1,2,2-tetrachloroethane. These compounds are often co-contaminants with TCP at contaminated sites as a result of their industrial application.^87,88^ Future research is needed to understand the identity of the highly reactive surface nucleophiles. The life cycle and techno-economic analysis of the proposed technology also warrant further investigation.

## Supporting Information Available

Details of the peak assignments for NMR; observed reaction constants, half-lives, and calculated activation energies of PLP-QA and PLP-py^+^; adsorption isotherms of SRNOM on PLP-QA and PLP-py^+^; control experiments of TCP degradation; (pseudo-first-order) degradation kinetics of TCP and Cl^–^ formation; reaction rate comparison among different technologies; allyl chloride degradation experiment (PDF)
