# Supplementary material for: Role of Nitrogenous Functional Group Identity in Accelerating 1,2,3-Trichloropropane Degradation by Pyrogenic Carbonaceous Matter (PCM) and Sulfide Using PCM-like Polymers
Source: Environ Sci Technol. 2024 Jun 7;58(24):10752–63. doi: 10.1021/acs.est.3c11010 (PMC11191598; doi:10.1021/acs.est.3c11010)
Supplement: Supplementary file 1 — es3c11010_si_001.pdf [file es3c11010_si_001.pdf]

SUPPORTING INFORMATION

Role of Nitrogenous Functional Group Identity in Accelerating 1,2,3-Trichloropropane Degradation by Pyrogenic Carbonaceous Matter (PCM) and Sulfide using PCM-like Polymers

Han Cao[1], Jingdong Mao[2], Paul G. Tratnyek[3], Wenqing Xu[1,*]

[1]Department of Civil and Environmental Engineering, Villanova University, Villanova, Pennsylvania 19085, United States

[2]Department of Chemistry and Biochemistry, Old Dominion University, Norfolk, Virginia 23529, United States

[3]OHSU/PSU School of Public Health, Oregon Health & Science University,

3181 SW Sam Jackson Park Road, Portland, OR 97239, United States

[*]Corresponding author:

Dr. Wenqing Xu

Email: wenqing.xu@villanova.edu

Phone: 610-519-8549

Supporting Information File for Environmental Science & Technology

Contents: 16 pages, 4 Tables, 12 Figures, and 14 References

Table S1. Peak assignments for NMR Spectra in Figure 1.

PLPs	$C_{Ar\text{-}O\text{-}R}$[1]	$C_{pyridine}$[2-4]	$C_{Ar\text{-}OH}$[5,6]	$C_{Ar\text{-}Br}$[7]	$C_{Ar\text{-}H}$[1,8]	C_{Ar}-C≡C-C_{Ar}[1,8]	$\text{-}C_{\equiv C}\text{-}$[1,8,9]	$C_{\text{-}OH}$[1]	C_{methyl}[1]
PLP-OH	--	--	152.3	139.2	132.8	125.5	92.8	--	--
PLP-QA	174.2	--	152.3	139.2	132.8	125.5	92.8	67.3	56.6
PLP-py	--	154.6, 143.6	--	139.2	133.6	125.5	92.8	--	--
PLP-py$^+$	--	ca. 150	--	139.2	133.6	125.5	ca. 85-100	--	51.4

Table S2. Observed reaction constant (k_{obs}), half-life ($t_{1/2}$), and calculated activation energy (E_a) of PLP-QA and PLP-py$^+$ calculated in Figure 3.

**PLP**	**T (°C)**	**k_{obs} (d^{-1})**	**Half-life ($t_{1/2}$, d)**	**Calculated E_a (kJ mol^{-1})**	**E_a of previous studies (kJ mol^{-1})**
**PLP-QA**	25	0.041±0.002	16.91±1.17	63.6±6.1	95.9±3.5[a,10]
	45	0.173±0.013	4.01±0.43		
	65	0.860±0.084	0.81±0.11		71.5~80[b,11]
**PLP-py$^+$**	5	0.136±0.008	5.13±0.41	51.9±4.9	
	25	0.717±0.077	0.98±0.15		61.9[c,12]
	45	2.269±0.063	0.31±0.01		

[a]Homogeneous abiotic reaction—hydrolysis
[b]Haloalkane Dehalogenases enzyme catalytic reaction
[c]Sonolysis in aqueous solution

Table S3. The obtained parameters of adsorption isotherm of SRNOM on PLP-QA and PLP-py$^+$ by non-linear fitting to the Freundlich Model.

**Sample**	**K_f [(mg g^{-1}) (L mg^{-1})$^{1/n}$]**	***n***
**PLP-QA**	0.24±0.13	0.77±0.11
**PLP-py$^+$**	0.15±0.11	0.74±0.13

Table S4. Conductivity of the PLPs.

**Material**	**σ (S/m)**
**PLP-OH**	≤0.01
**PLP-QA**	≤0.03
**PLP-py**	≤0.07
**PLP-py$^+$**	≤0.04
**CNT-OH**	≥100[1]

[1] Data was adopted from the vendor's website: https://www.cheaptubes.com/product/oh-functionalized-multi-walled-carbon-nanotubes-30-50nm/

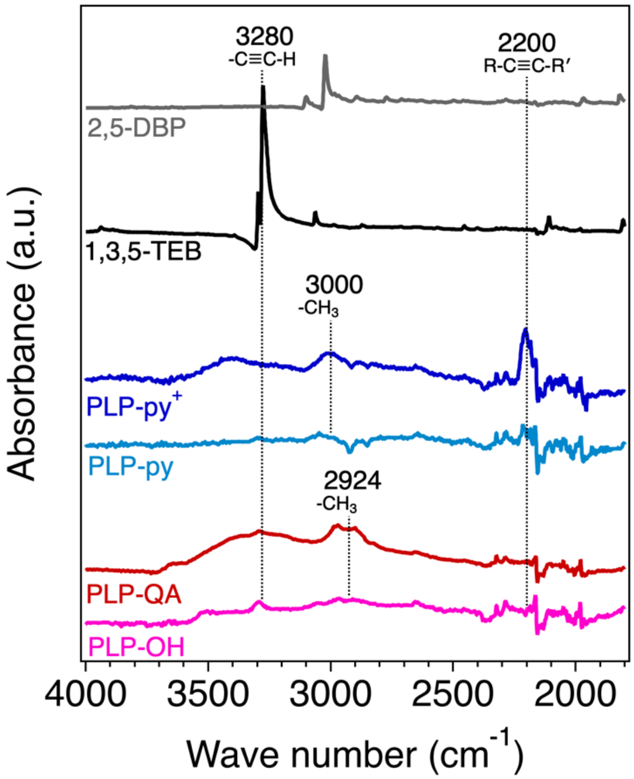

Figure S1. Fourier transform infrared (FTIR) spectroscopy of 2,5-dibromopyridine (2,5-DBP), 1,3,5-triethynylbenzene (1,3,5-TEB), PLP-py^{+}, PLP-py, PLP-QA and PLP-OH from top to bottom.

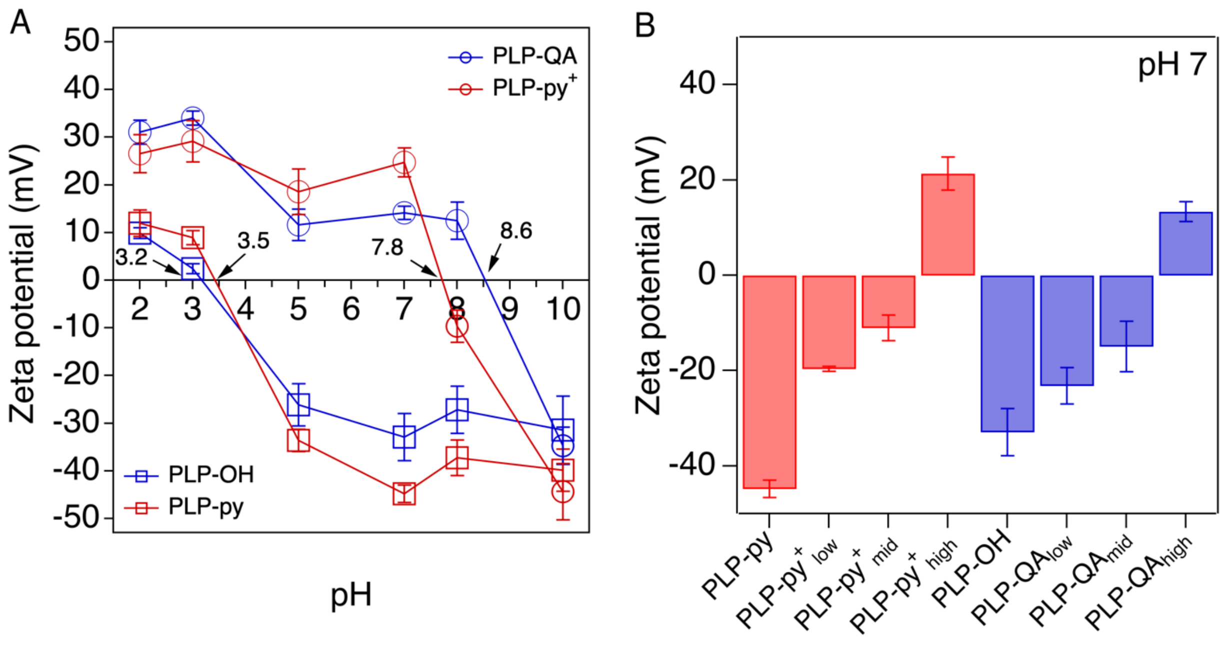

Figure S2. (**A**). Point-of-zero charge (pH_{pzc}) of PLP-OH, PLP-QA, PLP-py and PLP-py$^+$ used in this study. The PLP samples were prepared by dispersing each PLP in DI water, to which the pH was adjusted by adding HCl or NaOH to the desired values (i.e., pH 2, 3, 5, 7, 8 and 10). (**B**) Zeta potential of all PLPs measured at pH 7. The error bars were derived from triplicate measurements with 95% confidence level.

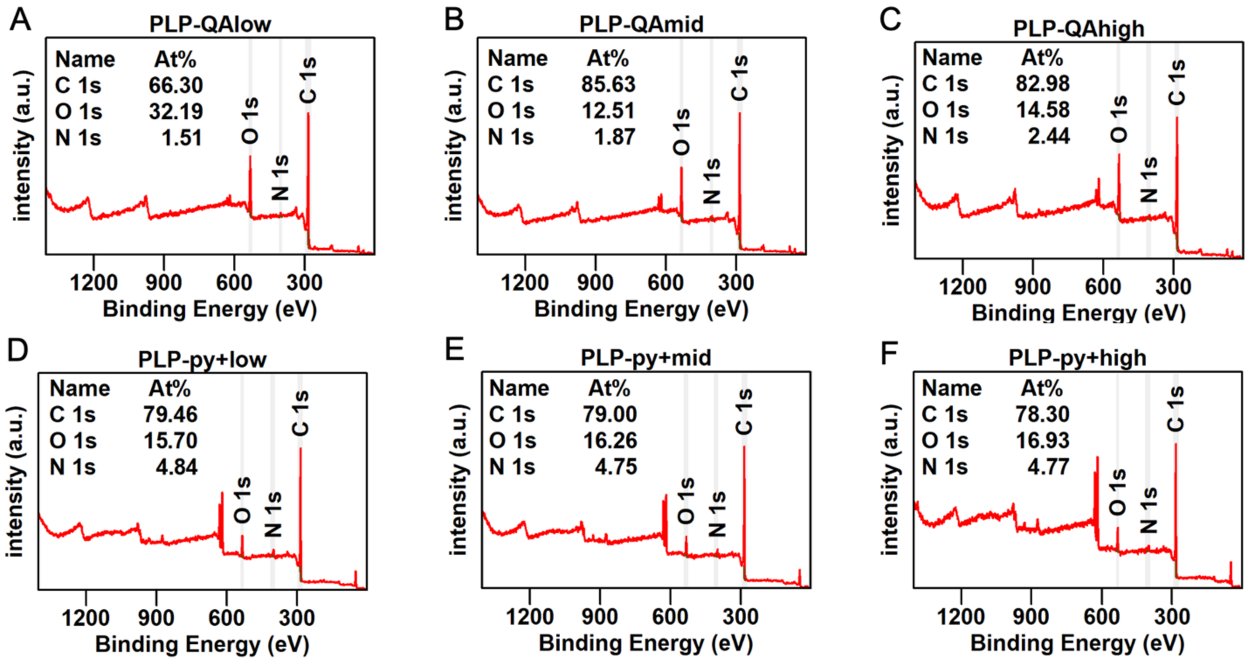

Figure S3. XPS survey of PLP-QA and py^+ at different density. (A) $PLP\text{-}QA_{low}$, (B) $PLP\text{-}QA_{mid}$, (C) $PLP\text{-}QA_{high}$, (D) PLP- py^+_{low}, (E) PLP- py^+_{mid}, and and (F) PLP- py^+_{high}.

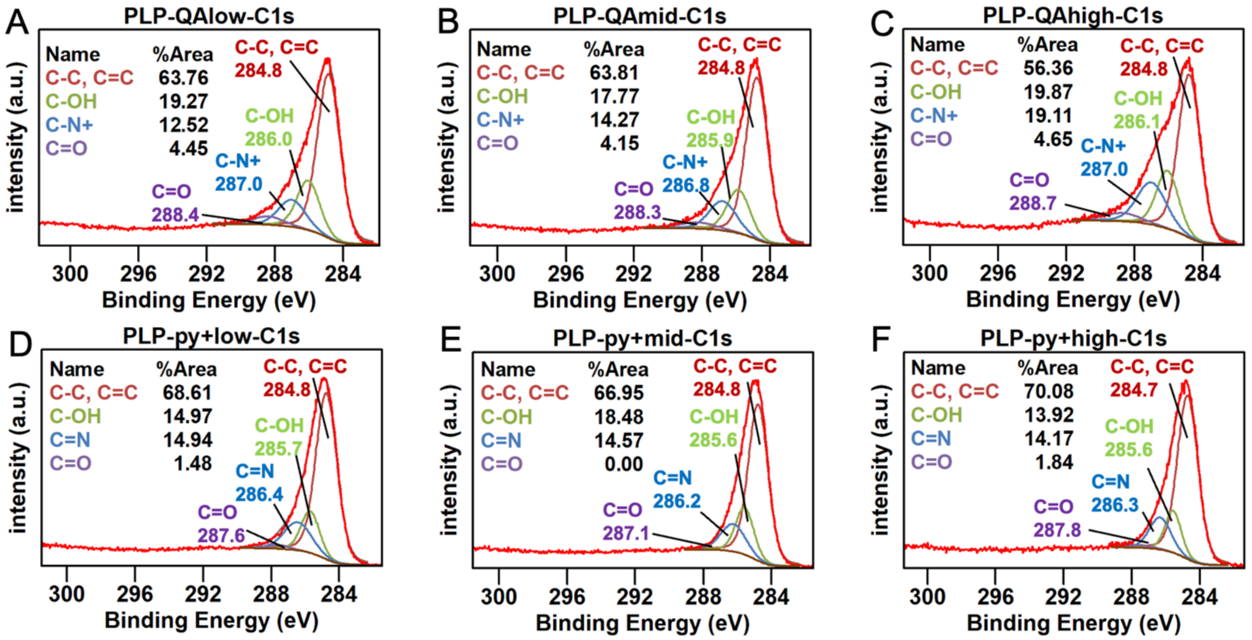

Figure S4. XPS C1s of PLP-QA and py^+ at different density. (A) PLP-QA_{low}, (B) PLP-QA_{mid}, (C) PLP-QA_{high}, (D) PLP- py^+_{low}, (E) PLP- py^+_{mid}, and (F) PLP- py^+_{high}.

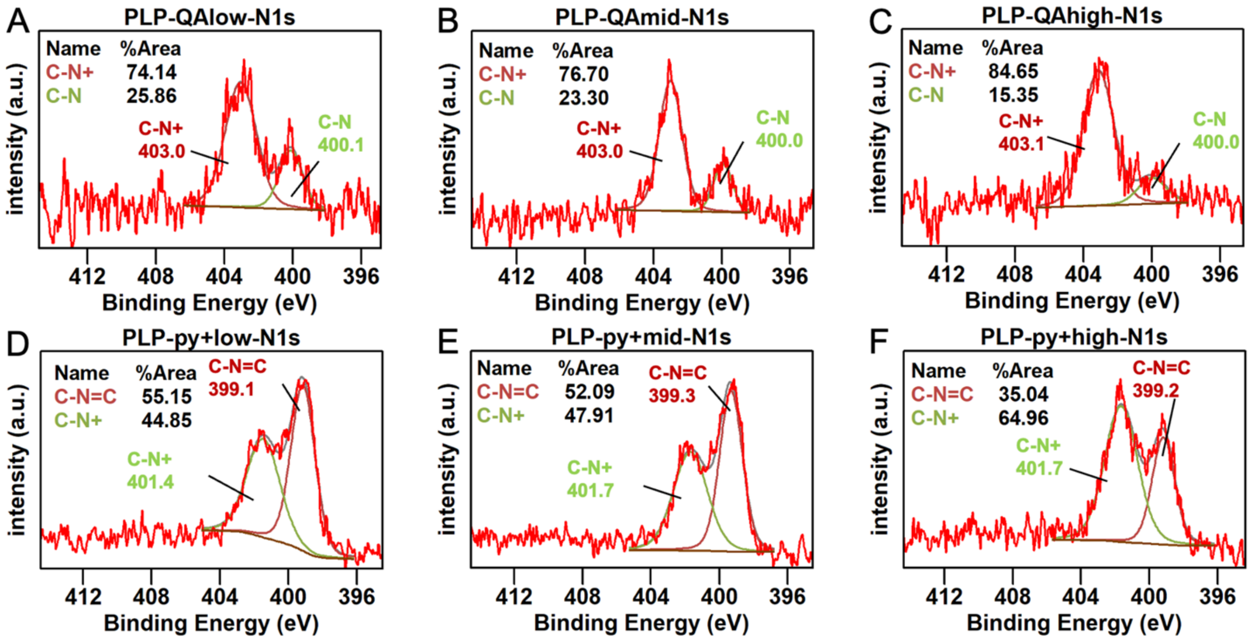

Figure S5. XPS N1s of PLP-QA and py$^+$ at different density. (A) PLP-QA$_{low}$, (B) PLP-QA$_{mid}$, (C) PLP-QA$_{high}$, (D) PLP- py$^+_{low}$, (E) PLP- py$^+_{mid}$, and (F) PLP- py$^+_{high.}$

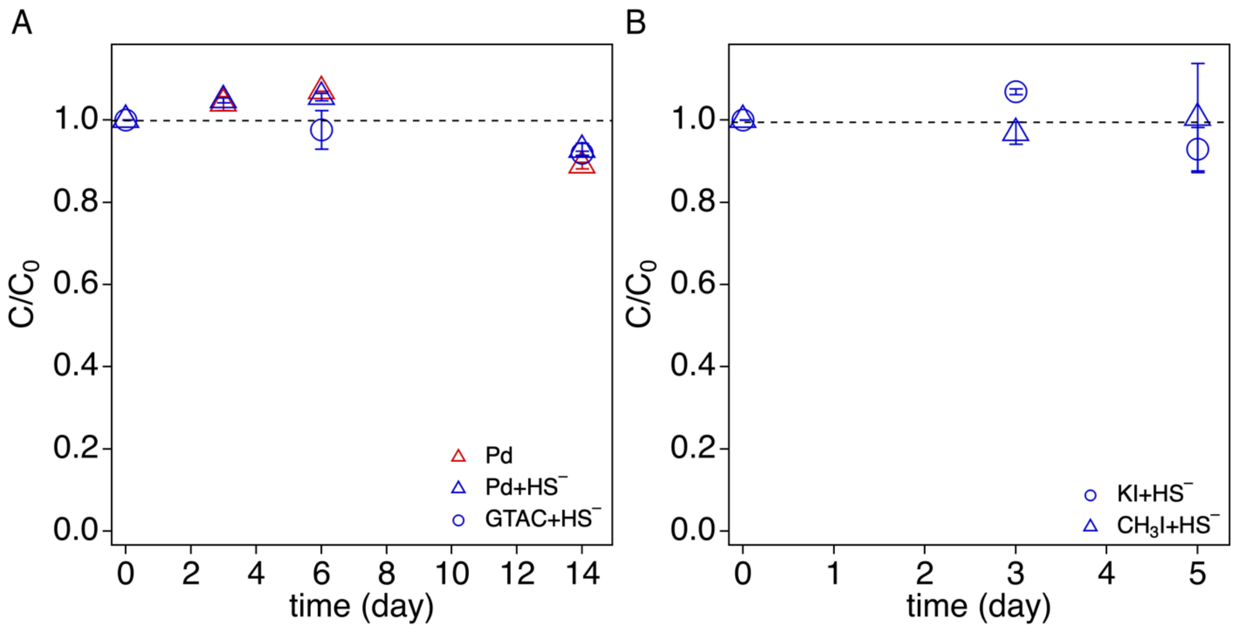

Figure S6. (**A**) 1,2,3-Trichloropropane (TCP) degradation in the presence of 5 mM *TOT*HS and 0.07 g·L^{-1} palladium catalyst ($Pd(PPh_3)_4$) and 0.7 g·L^{-1} glycidyltrimethylammonium chloride (GTAC). Examination of TCP degradation in the presence of 0.07 g·L^{-1} Pd alone without adding sulfide was also included for comparison. (**B**) TCP degradation in the presence of 5 mM *TOT*HS and 0.7 g·L^{-1} CH_3I and 0.7 g·L^{-1} KI. The error bars were derived from triplicate samples with 95% confidence level.

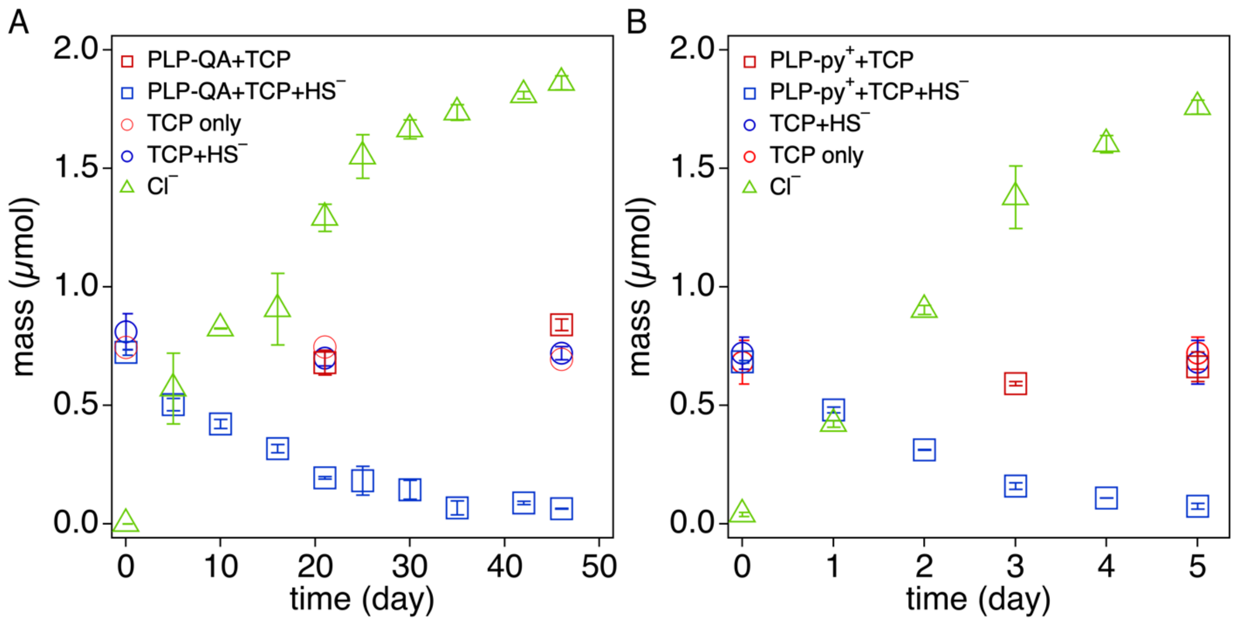

Figure S7. 1,2,3-Trichloropropane (TCP) degradation in the presence of 5mM sulfide at pH 7 (20 mM phosphate buffer) and 0.7 g·L^{-1} of (**A**) quaternary ammonium (QA) grafted PCM-like polymer (PLP-QA) and (**B**) pyridinium cation (py^+) grafted PLP (PLP-py^+) under room temperature (25 °C). The error bars were derived from triplicate samples with 95% confidence level.

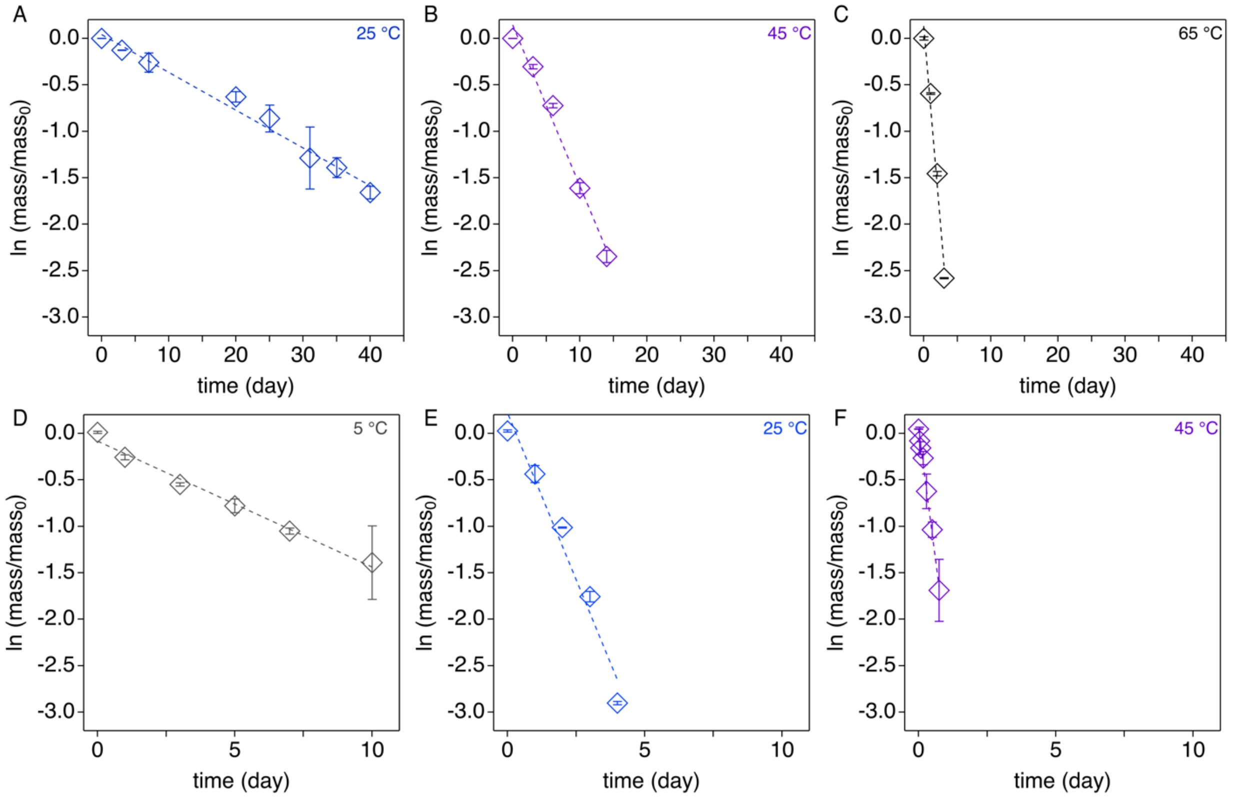

Figure S8. Pseudo-first order degradation kinetics of 1,2,3-Trichloropropane (TCP) in the presence of 5mM sulfide and 0.7 g·L^{-1} of (**A**), (**B**) and (**C**) PLP-QA or of (**D**), (**E**) and (**F**) PLP-py+ at pH 7 (20 mM phosphate buffer) under 5, 25 and 45 °C. The error bars were derived from triplicate samples with 95% confidence level.

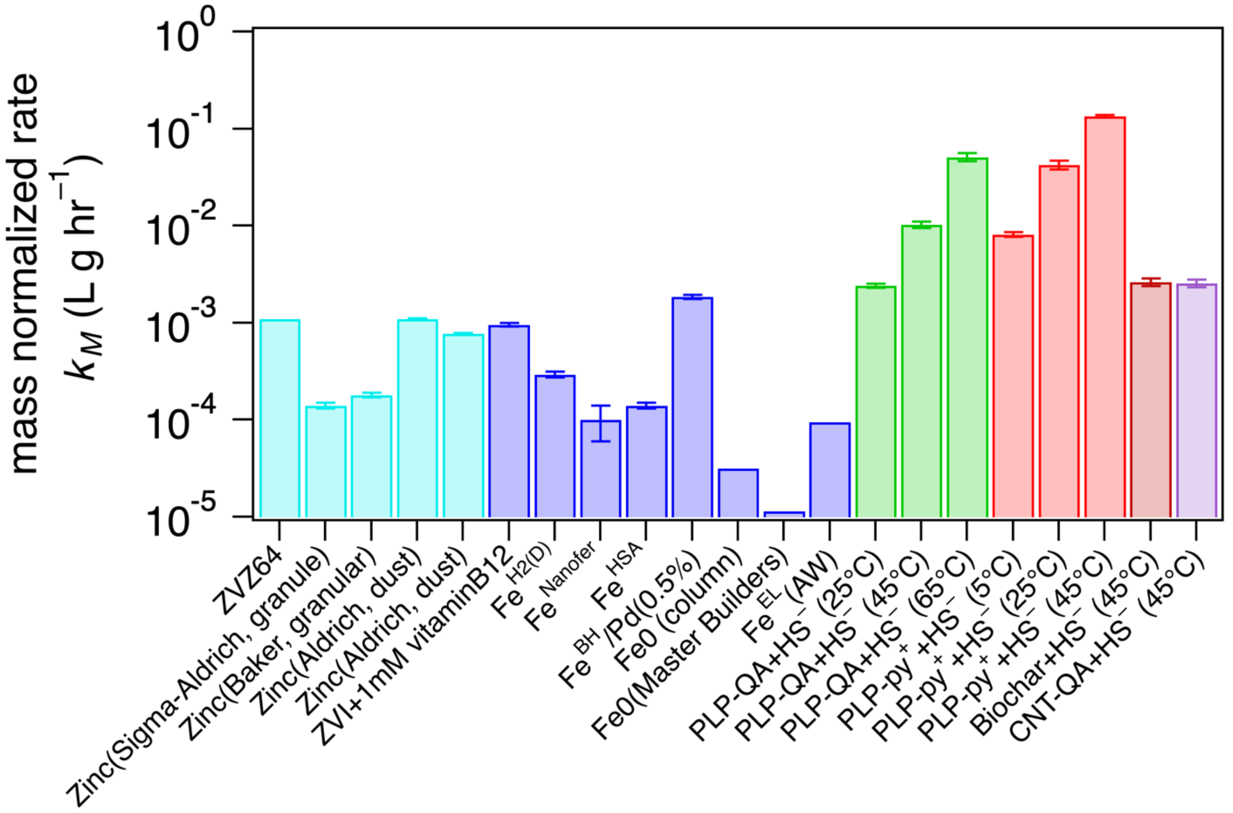

Figure S9. Mass normalized observed reaction rate constants (k_M) of TCP decay by different technologies that involve solid. Zero-valent zinc (ZVZ, cyan) and zero-valent iron (ZVI, purple) data are adopted from Sarathy et al.[13] and Salter-Blanc and Tratnyek.[14] The abbreviations for the different ZVZ's and ZVI's are given in the original sources. Other data are from this study. The error bars were either directly adopted from the referenced studies, or derived from triplicate samples with 95% confidence level (our study).

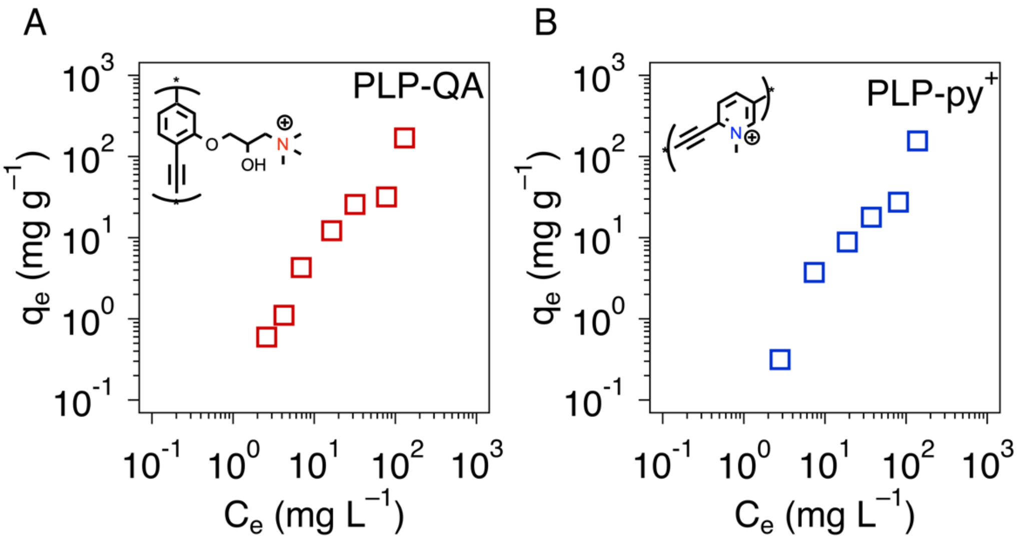

Figure S10. Adsorption isotherm of SRNOM on (**A**) PLP-QA and (**B**) PLP-py$^+$. The data was fitted to the Freundlich model (i.e., $q_e = K_f \bullet C_e^{1/n}$) and the obtained fitting parameters are: K_f for PLP-QA and PLP-py$^+$ were 0.24±0.13 and 0.15±0.11 [(mg g^{-1}) (L mg^{-1})$^{1/n}$], respectively. The calculated n for PLP-QA and PLP-py$^+$ were 0.77±0.11 and 0.74±0.13, respectively.

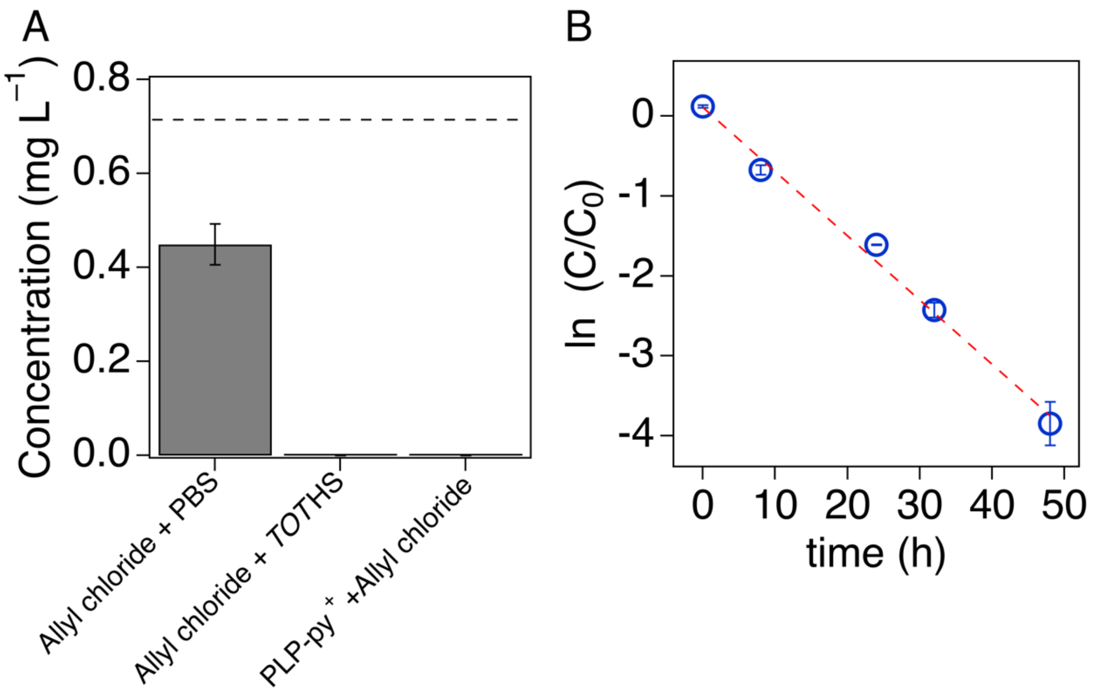

Figure S11. (**A**) The total mass of allyl chloride in water (dashed line), PBS, 5mM *TOT*HS, and PLP-py$^+$ (0.7g L^{-1}) at pH 7 after 5 days. The extraction efficiency of TCP from PLP was above 95%. (**B**) Allyl chloride degradation by 5mM *TOT*HS in the absence of PLPs at pH 7 under 25 °C. The calculated k_{obs} was 0.08±0.01 h^{-1}, corresponding to a half-life $t_{1/2}$ of 8.7±0.6 h for allyl chloride degradation. The error bars were derived from triplicate samples with 95% confidence level.

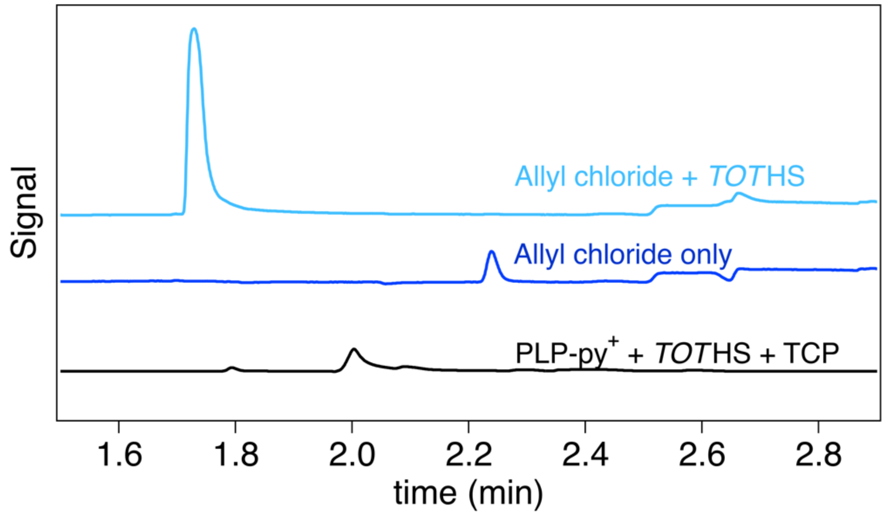

Figure S12. Chromatogram of gas chromatography with an electron capture detector (GC-ECD) for allyl chloride only (blue), and with 5 mM *TOT*HS after 5 days (cyan). The retention time of allyl chloride and the new peak is 2.24 and 1.73 min, respectively.

Reference:

1. Li, Z.; Jorn, R.; Samonte, P. R. V.; Mao, J.; Sivey, J. D.; Pignatello, J. J.; Xu, W., Surface-catalyzed hydrolysis by pyrogenic carbonaceous matter and model polymers: An experimental and computational study on functional group and pore characteristics. *Applied Catalysis B: Environmental* **2022,** *319*, 121877. doi.org/10.1016/j.apcatb.2022.121877.

2. Meng, F.; Bi, S.; Sun, Z.; Jiang, B.; Wu, D.; Chen, J.-S.; Zhang, F., Synthesis of Ionic Vinylene-Linked Covalent Organic Frameworks through Quaternization-Activated Knoevenagel Condensation. *Angewandte Chemie International Edition* **2021,** *60*, (24), 13614-13620. doi.org/10.1002/anie.202104375.

3. Yuan, K.; Zhuang, X.; Fu, H.; Brunklaus, G.; Forster, M.; Chen, Y.; Feng, X.; Scherf, U., Two-Dimensional Core-Shelled Porous Hybrids as Highly Efficient Catalysts for the Oxygen Reduction Reaction. *Angewandte Chemie International Edition* **2016,** *55*, (24), 6858-6863. doi.org/10.1002/anie.201600850.

4. Qi, J.-X.; Zhang, C.-R.; Chen, X.-J.; Yi, S.-M.; Niu, C.-P.; Liu, J.-L.; Zhang, L.; Liang, R.-P.; Qiu, J.-D., 3D Ionic Olefin-Linked Conjugated Microporous Polymers for Selective Detection and Removal of TcO4–/ReO4– from Wastewater. *Analytical chemistry* **2022,** *94*, (30), 10850-10856. 10.1021/acs.analchem.2c01932.

5. Li, B.; Gong, R.; Wang, W.; Huang, X.; Zhang, W.; Li, H.; Hu, C.; Tan, B., A New Strategy to Microporous Polymers: Knitting Rigid Aromatic Building Blocks by External Cross-Linker. *Macromolecules* **2011,** *44*, (8), 2410-2414. 10.1021/ma200630s.

6. Zhang, C.; Yang, X.; Zhao, Y.; Wang, X.; Yu, M.; Jiang, J.-X., Bifunctionalized conjugated microporous polymers for carbon dioxide capture. *Polymer* **2015,** *61*, 36-41. doi.org/10.1016/j.polymer.2015.01.072.

7. Dawson, R.; Laybourn, A.; Clowes, R.; Khimyak, Y. Z.; Adams, D. J.; Cooper, A. I., Functionalized Conjugated Microporous Polymers. *Macromolecules* **2009,** *42*, (22), 8809-8816. 10.1021/ma901801s.

8. Li, Z.; Mao, J.; Chu, W.; Xu, W., Probing the Surface Reactivity of Pyrogenic Carbonaceous Material (PCM) through Synthesis of PCM-Like Conjugated Microporous Polymers. *Environmental Science & Technology* **2019,** *53*, (13), 7673-7682. 10.1021/acs.est.9b01772.

9. Hua, C.; Chan, B.; Rawal, A.; Tuna, F.; Collison, D.; Hook, J. M.; D'Alessandro, D. M., Redox tunable viologen-based porous organic polymers. *Journal of Materials Chemistry C* **2016,** *4*, (13), 2535-2544. 10.1039/C6TC00132G.

10. Pagan, M.; Cooper, W. J.; Joens, J. A., Kinetic studies of the homogeneous abiotic reactions of several chlorinated aliphatic compounds in aqueous solution. *Applied Geochemistry* **1998,** *13*, (6), 779-785. doi.org/10.1016/S0883-2927(98)00005-5.

11. Marques, S. M.; Dunajova, Z.; Prokop, Z.; Chaloupkova, R.; Brezovsky, J.; Damborsky, J., Catalytic Cycle of Haloalkane Dehalogenases Toward Unnatural Substrates Explored by Computational Modeling. *Journal of chemical information and modeling* **2017,** *57*, (8), 1970-1989. 10.1021/acs.jcim.7b00070.

12. Lim, M. H.; Kim, S. H.; Kim, Y. U.; Khim, J., Sonolysis of chlorinated compounds in aqueous solution. *Ultrasonics Sonochemistry* **2007,** *14*, (2), 93-98. doi.org/10.1016/j.ultsonch.2006.03.003.

13. Sarathy, V.; Salter, A. J.; Nurmi, J. T.; O'Brien Johnson, G.; Johnson, R. L.; Tratnyek, P. G., Degradation of 1,2,3-Trichloropropane (TCP): Hydrolysis, Elimination, and Reduction by Iron and Zinc. *Environmental Science & Technology* **2010,** *44*, (2), 787-793. 10.1021/es902595j.

14. Salter-Blanc, A. J.; Tratnyek, P. G., Effects of Solution Chemistry on the Dechlorination of 1,2,3-Trichloropropane by Zero-Valent Zinc. *Environmental Science & Technology* **2011,** *45*, (9), 4073-4079. 10.1021/es104081p.
